# Seed the Difference: QTL Mapping Reveals Several Major Loci for Seed Size in *Cannabis sativa* L.

**DOI:** 10.3390/plants14243853

**Published:** 2025-12-17

**Authors:** Stephen Eunice Manansala-Siazon, Paolo Miguel Siazon, Erwin Tandayu, Lennard Garcia-de Heer, Adam Burn, Qi Guo, Jos C. Mieog, Tobias Kretzschmar

**Affiliations:** 1 Faculty of Science and Engineering, Southern Cross University, Lismore, NSW 2480, Australia; s.siazon.10@student.scu.edu.au (S.E.M.-S.);; 2 Agriculture Victoria, 5 Ring Road, Bundoora, VIC 3083, Australia

**Keywords:** *Cannabis sativa*, hempseed, QTL mapping, seed traits, plant height, inflorescence structure, trichome density, molecular markers, plant breeding

## Abstract

*Cannabis sativa* L. has been cultivated for millennia as a source of food and fibre. Increasing demand for functional foods has renewed interest in *C. sativa* seeds (hempseeds), which are rich in essential fatty acids and amino acids. However, a near-global moratorium on *C. sativa* cultivation and research throughout most of the 20th century has delayed crop improvement using modern breeding approaches. As a result, genetic loci contributing to key agronomic traits, including with respect to maximizing yield as a seed crop, remain largely unknown. In this study, a feminized segregating F_2_ mapping population, derived from a tall parent with spacious inflorescences and large seeds and a short-stature parent with compact inflorescences and small seeds, was phenotyped for key seed and agronomic traits related to yield. A mid-density Single Nucleotide Polymorphism (SNP) genotyping panel was used to generate a genetic linkage map of 291.5 cM with 455 SNPs. Quantitative Trait Locus (QTL) mapping identified major loci for hundred-seed weight—*qHSW3*, 26.59 percent variance explained (PVE), seed volume—*qSV1*, 33.24 PVE, and plant height—*qPH9*, 46.99 PVE. Our results provide novel target regions, associated molecular markers, and candidate genes for future breeding efforts to improve *C. sativa.*

## 1. Introduction

*Cannabis sativa* L. is a versatile crop species that has long been cultivated for food, fibre and medicine [1,2,3]. Today, industrial hemp is typically grown in broadacre settings to harvest its seed and/or fibres (bast and hurd), while drug-type cannabis, or marijuana, is mainly cultivated in protected cropping systems for medicinal and recreational purposes. The industrial hemp and medicinal cannabis industries are strictly segregated and regulated based on specific thresholds for THC (Δ^9^-tetrahydrocannabinol) content—the cannabinoid responsible for psychoactive effects [4].

The earliest recorded utilization of *C. sativa* was for its nutrients and fibres [3,5,6]. Its seeds serve as a food source, which can be consumed either whole or processed into oils, flours or protein extracts [7,8,9]. Containing around 35–36% of oil, 24–26% protein, and 27–29% carbohydrate [2], hempseeds can be regarded as both an oilseed and a protein crop. Hempseed oil contains high amounts of polyunsaturated fatty acids (PUFAs) and is particularly rich in the essential fatty acids linoleic acid (LA) and alpha-linolenic acid (ALA), which are at an optimal ratio for human nutrition [8,10]. Dietary inclusion of hempseed oil can contribute to lowered cholesterol levels [7,11] and can have positive effects on plasma fatty acid profiles [12]. Hempseed protein contains all essential amino acids and is particularly rich in arginine, an amino acid with potential hypotensive effects [13]. Because of these nutritional and nutraceutical qualities, hempseed and derived products qualify as a functional food and have seen increasing demand among health-conscious consumers [9]. However, the high price of hempseed products is limiting large-scale adoption into mainstream diets [14].

Part of the reason for the high cost of hempseed can be attributed to a lack of targeted development of hemp as a high-yielding oilseed crop. Properly developed, it could provide similar oil and protein yields as traditional oilseeds (e.g., canola or soy) or protein crops (e.g., pulses). To fully exploit its potential as a nutritional resource, targeted breeding that focuses on improving seed quality traits and yield is paramount [15]. Targeted breeding approaches rely on the knowledge of the genetic basis for economically important traits, which can be identified through quantitative trait loci (QTL) studies [16]. Molecular markers can then be developed for QTLs, increasing the efficiency of breeding programmes [16]. The absence of identified QTLs for seed traits currently limits progress in breeding cultivars specialized for seed production. Seed size, which contributes to both yield and nutritional value, is considered a key trait in this context [17].

**Figure 1:**
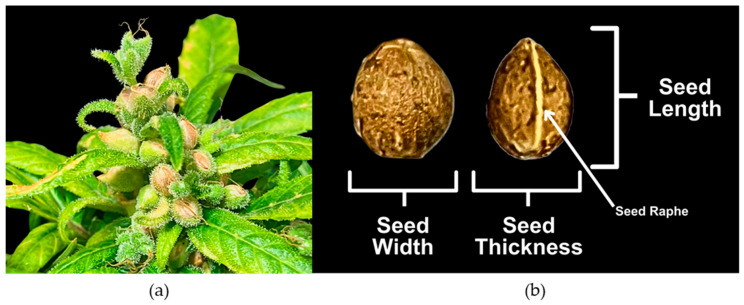
Morphology and structure of *C. sativa* seeds. (**a**) Matured hempseeds in planta approximately 12 weeks post pollination; (**b**) Hempseed structure showing seed length, seed width, and seed thickness as characterized by seed raphe.

Hemp plants are mainly dioecious and dimorphic, with monoecious plants rarely occurring naturally, but are often favoured in a seed production context [1]. Staminate plants have large, exposed, loose, axillary, cymose panicles, in contrast to the small, obscure, congested, axillary, spicate cymes of pistillate plants [18]. Seeds develop in an enclosed bract studded by glandular trichomes (Figure 1a) [1,19]. These glandular trichomes are also the site of cannabinoid biosynthesis, and trichome density has been shown to be an important factor in overall cannabinoid accumulation [20]. Hempseeds, technically achenes, are ellipsoid in shape and slightly flat on the side, with a raphe distinguishing their width from their thickness (Figure 1b) [18]. They are brown or grey in colour, with some exhibiting mosaic patterns [1,18]. During seed development, maternal tissues, particularly the sporophytic integuments, gradually differentiate into the seed coat [21,22]. As an achene, a dry one-seeded fruit similar to Arabidopsis, hempseed contains a pericarp and testa that are also derived from maternal tissues [23].

Seed size is determined by a range of factors—involving both maternal and zygotic controls, as well as interactions between them [21,24]. While nutrition and environmental factors play a role in seed size, this complex multigenic trait is largely controlled by genetic factors [24]. In other plant species, various pathways that regulate the growth of maternal tissues have been widely studied [21,24]. Although seed size across species varies greatly as it is an adaptive characteristic, there are commonalities in the pathways that are involved in its regulation [21]. They have been studied and reviewed in great depth [21,25] and include (i) phytohormonal regulation (e.g., auxins, cytokinins, brassinosteroids and jasmonates); (ii) transcriptional regulation; (iii) post-transcriptional and post-translational regulation (e.g., phosphorylation and ubiquitination); as well as (iv) epigenetic regulation (e.g., DNA methylation and demethylation). Major dicot seed crops such as *C. sativa*, e.g., soybean and canola, have similar seed development programmes to that of *Arabidopsis* [22].

Seed size primarily contributes to seed weight and is an indicator of yield, making it a key target for conventional crop breeding [25]. While it is considered a valuable adaptive character that contributed to evolutionary success across plant species, artificial selection resulting from the domestication of cultivated crops has significantly altered seed size relative to its wild progenitors [21,24,25]. However, seed size and weight within species still show considerable diversity between, and sometimes even within, cultivars such as in soybean and rapeseed [25,26]. Differences in hempseed size and weight can affect post-harvest processing, including seed sorting, dehulling, and the suitability for other downstream processes such as milling and oil pressing [27,28]. In hemp, seed weight further has a strong correlation with oil and fatty acid content—and consequently seed quality [27].

Finally, plant architecture affects the yields and harvestability of hempseed. Ideally, hempseed varieties such as Finola have heights less than 1.5 m and show little to no branching, allowing efficient harvesting for seeds [29]. Low-branching, short-stature plants with compact apical inflorescences and large, uniform seeds can serve as a model for seed production type hemp. Breeding for such varieties can be fast-tracked through marker-assisted selection, provided that robust major QTLs for the corresponding target traits have been characterized.

In this study, we identified several major QTLs for seed traits, including hundred seed weight, seed length, seed width, and seed volume in hemp through mapping in a biparental cross between a dual-purpose hemp accession, SI-1, and Syrian, a landrace accession. Major QTLs for agronomic traits such as plant height, longest branch, and internode count were also found. Importantly, there was no significant phenotypic correlation between seed traits and agronomic traits, suggesting that small plants with large seeds can be effectively selected for.

## 2. Results

### 2.1. Parental Selection

**Figure 2:**
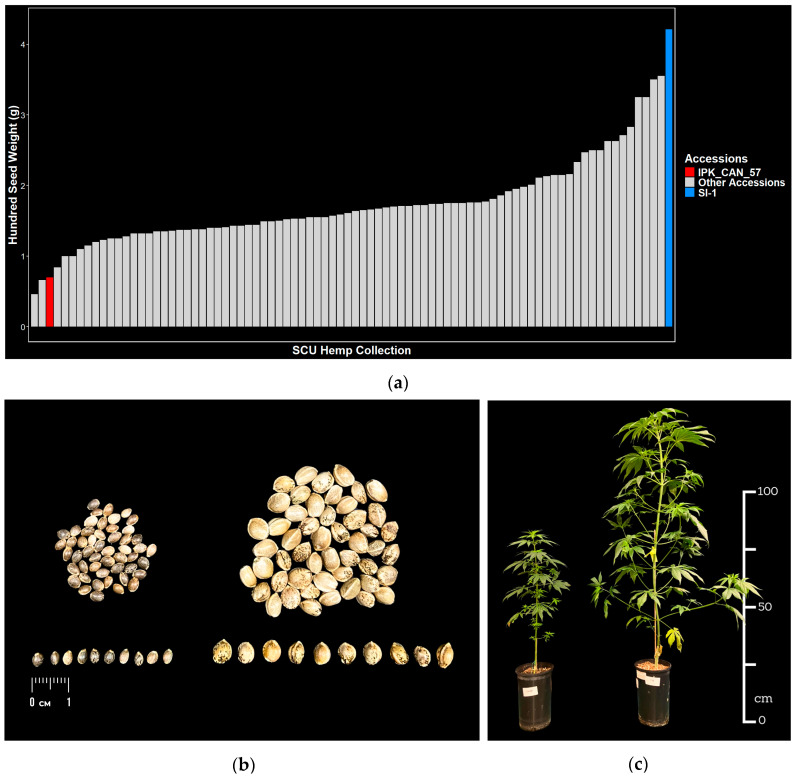
**Phenotypic contrasts between selected parental accessions.** (**a**) Hundred seed weight of 84 hemp accessions highlighting IPK_CAN_57 (red) and SI-1 (blue); (**b**) Seeds of IPK_CAN_57 (left, n = 50) and SI-1 (right, n = 50); (**c**) Plant architecture of vegetative IPK_CAN_57 (**left**) and SI-1 (**right**) at 7 weeks after germination.

Seeds from a diverse panel of 84 hemp accessions from 14 countries (Table S1) are assessed for weight and size. Average hundred seed weights range from 0.5 g to 4.2 g (Figure 2a, Table S1), while average seed lengths and widths range from 3.1 to 5.9 mm and 2.2 to 4.5 mm, respectively (Figure S1, Table S1). The largest and heaviest seed is derived from SI-1, a tall, late maturing variety from China, while amongst the lightest seed is IPK_CAN_57, a Syrian landrace of small stature, which had the smallest seed of all accessions assessed (Figure 2a,b and Figure S1). In addition to seed size, SI-1 and IPK_CAN_57 also show distinct phenotypes for plant architecture (Figure 2c). At 7 weeks after germination, SI-1 grew to a height of approximately 115 cm while IPK_CAN_57 was only 65 cm tall. SI-1 was wider (~75 cm) and more branched than IPK_CAN_57 (~30 cm) and had more elongated internodes (Figure 2c).

Genotyping of the diverse panel reveals that SI-1 and IPK_CAN_57 cluster into distinct nodes (Figure S2, Table S2). While SI-1 clusters with other germplasm of Chinese origin, IPK_CAN_57 clusters with germplasm from Turkey, Italy, and Argentina (Figure S2).

Based on contrasting seed phenotypes, plant height, and genetic distance, SI-1 and IPK_CAN_57 are selected as parents for an F_2_ biparental mapping population with a focus on identifying QTLs related to seed size (Figure S3).

### 2.2. Predicting Seed Thickness and Volume

While two-dimensional scans of seeds allow for precise measurements of seed length and width, seed thickness (Figure 1b) could not be captured by image-based analysis. From manual measurements of seed length, width, and thickness of SI-1 (n = 25), IPK_CAN_57 (n = 25), and F_2_-derived seeds (n = 510), the relationships of thickness with width and length were compared, with coefficients of determination (R^2^) equal to 0.85 and 0.74, respectively (Table S3, Figure S4). Seed thickness was, therefore, predicted through a linear regression model derived from seed width values. The model showed that seed width was a significant predictor of seed thickness (*p*-value < 2 × 10^−16^) and that for every unit increase in seed width, seed thickness increases by approximately 0.81 units (Table S4). Seed volume was determined using the predicted seed thickness, the measured seed length and width, and the coefficient of seed volume from a previously published formula [30].

### 2.3. Seed Phenotypes Across Generations

**Figure 3:**
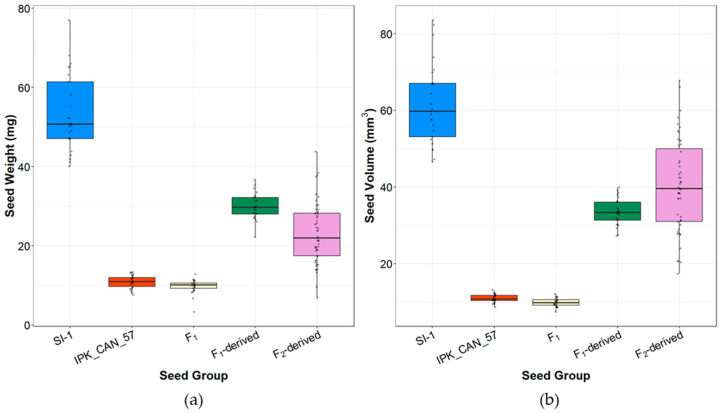
Comparative seed phenotypes across parental and derived generations. (**a**) Measured seed weight and (**b**) computed seed volume of SI-1 (n = 25), IPK_CAN_57 (n = 25), F_1_ (n = 25), F_1_-derived (n = 25), and F_2_-derived seeds (n = 51).

Individual seed weights, volumes, and sizes were compared for parents (SI-1 and IPK_CAN_57), F_1_, F_1_-derived (F_2_) and F_2_-derived seed (Figure 3 and Figure S5a, Table S5). SI-1 seeds had median weights of 53.4 mg at median volumes of 61.7 mm^3^, while IPK_CAN_57’s seeds were about 5-fold lighter with 11.0 mg at 10.9 mm^3^ (Table S6). F_1_ seeds had a median weight of 9.7 mg at 9.9 mm^3^ in volume, which was not significantly different from that of the female parent IPK_CAN_57. F_1_-derived seeds showed intermediate seed phenotypes (29.9 mg at 33.5 mm^3^) that were significantly different from both parental seeds and the F_1_ seeds (Table S6). A similar trend was observed for F_2_-derived seeds (23.1 mg at 40.3 mm^3^). F_1_- and F_2_-derived seeds were similar in seed volume but significantly different in seed weight (Table S6). Additionally, F_2_-derived seeds exhibit a much more pronounced range in both weight (6.9–43.8 mg) and volume (17.3–67.8 mm^3^) as compared to the F_1_-derived seed (22.2–36.7 mg and 27.3–39.9 mm^3^) (Figure 3). Plotting seed weight against seed volume for all the different seed groups showed a linear relationship between the two (R^2^ = 0.72, *p*-value < 0.05, Figure S5b).

Across the 147 F_2_-derived seed lots, hundred-seed weight ranged from 0.7 to 4.4 g and showed a high variation, CV (coefficient of variation) of 28.6%, and a normal distribution within the population (Figure S6, Table 1 and Table S7). Seed length ranged from 3.9 mm to 6.0 mm, and seed width ranged from 3.4 mm to 5.4 mm. Both show low CVs of 7.7% and 8.4%, respectively, and were normally distributed (Table 1, Figure S6c,d). The predicted seed volume and seed density had high CVs of 23.3% and 26.3% with ranges from 18.5 to 66.4 mm^3^ and 0.3 to 1.1 mg/mm^3^, while showing high frequencies at 30–40 mm^3^ and 0.9 mg/mm^3^ (Table 1, Figure S6b,e).

### 2.4. Seed and Agro-Morphological Phenotypes of the F_2_ Population

In addition to quantifying seed traits, the F_2_ population was assessed for agro-morphological traits (Table S7, Figure S7). The observed phenotypes were highly varied, with a minimum CV of 23.0% for stem diameter and a maximum of 59.1% for trunk length (Table 1). Plant heights ranged from 21 cm to 210 cm with a CV of 43.6%. The number of internodes varied from 5 to 23 (at an average of 10), while average internode lengths per plant ranged from 2.3 to 15.1 cm (Table 1, Figure S7a,c,d). The longest branch observed was 90 cm, while the highest plant width was 89 cm (Table 1, Figure S7e,f). Stem diameter ranges from 3.0 to 13.4 mm, with a CV of 23.0% and pith cavity diameter had a CV of 48.1%, with a maximum value of 6.0 mm, and some plants have no pith cavity at all (Table 1, Figure S7g,h). Inflorescence compactness and trichome density have CVs of 32.7% and 55.3%, respectively, with most plants having a score of two for inflorescence compactness and 1–2 for trichome density (Table 1, Figure S7i,j).

### 2.5. Correlation Among Phenotypes

**Figure 4:**
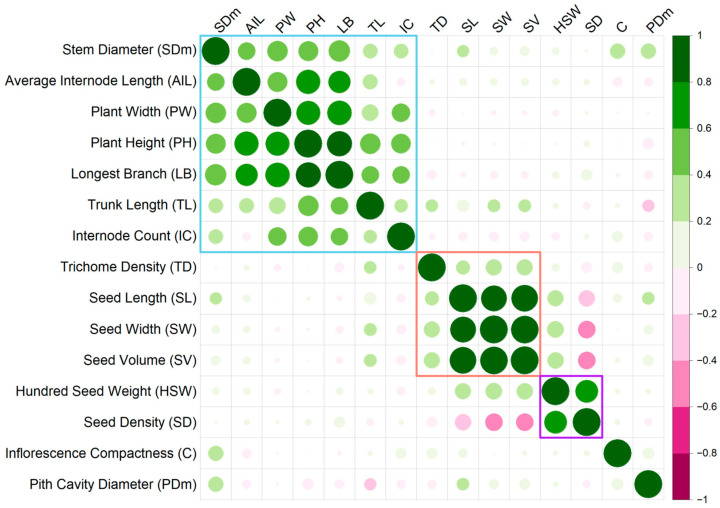
Pearson correlation matrix of seed traits and agronomic traits phenotyped in this study (*p*-value < 0.05), where green represents positive correlations and pink represents negative correlations. Coloured squares indicate positively correlated trait groups: blue—plant agro-morphological phenotypes, red—seed dimension traits, purple—seed mass-related traits. Correlation coefficients and *p*-values for all comparisons are listed in Table S8.

Correlation analysis suggests a clustering of positive correlations among most seed traits (Figure 4, Table S8). Seed length, width, and volume showed very strong positive correlations among each other (Pearson correlation coefficient (*r*) = 0.88 to 0.99) and a weaker positive correlation with trichome density (*r* = 0.27 to 0.34). Correlation between hundred-seed weight and seed density was also strong (*r* = 0.68). However, while hundred-seed weight showed positive correlations with seed volume (*r* = 0.35) and size (length *r* = 0.35; width *r* = 0.37), seed density was negatively correlated with volume (*r* = −0.41) and size (length *r* = −0.36; width *r* = −0.41).

Similarly, agro-morphological traits formed a positively correlated cluster (Figure 4, Table S8). Plant height had high positive correlations with other agro-morphological traits, i.e., longest branch (*r* = 0.83), average internode length (*r* = 0.77), plant width (*r* = 0.75), trunk length (*r* = 0.54), stem diameter (*r* = 0.53), and internode count (*r* = 0.50). All correlations were significant (*p*-value < 0.05). These traits were also positively correlated with each other (apart from average internode length and count), with Pearson correlation coefficients ranging from 0.24 to 0.83 for significant correlations.

In contrast, agro-morphological traits did not have significant correlations with seed traits. Inflorescence compactness and pith cavity diameter showed no significant correlations with the other traits, except for positive correlations with stem diameter (*r* = 0.29 and 0.31, respectively).

### 2.6. Genotype Data and QTL Mapping

**Figure 5:**
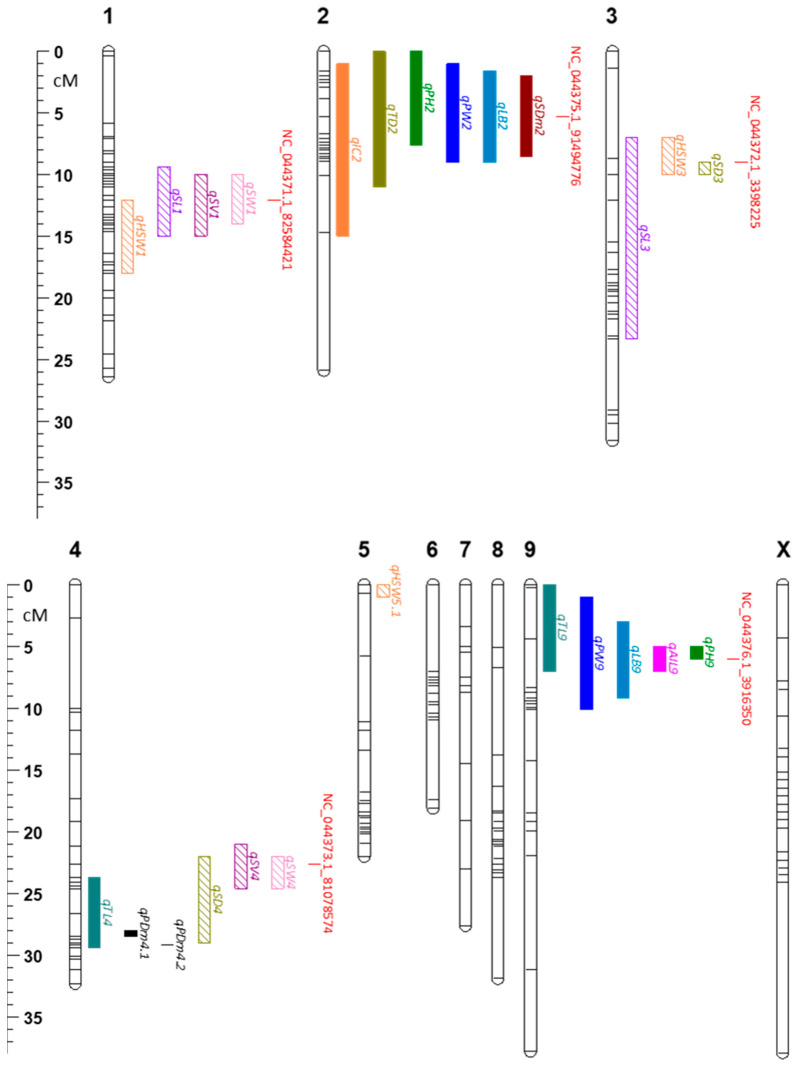
Genetic linkage map and distribution of major QTLs identified in the F_2_ mapping population. The map was generated from 222 F_2_ individuals genotyped using the HASCH panel and mapping positions of major QTLs (LOD exceeds permutation-derived significance threshold (α = 0.05 with Percent Variance Explained > 10%) seed traits (striped bars) and plant agronomic traits (solid bars) identified in this study. Peak markers that are common for multiple QTLs and their positions are also shown in red. Abbreviations: *qAIL*—average internode length; *qHSW*—hundred seed weight; *qIC*—internode count; *qLB*—longest branch; *qPH*—plant height; *qPW*—plant width; *qSD*—seed density; *qPDm*—stem pith cavity diameter; *qSL*—seed length; *qSV*—seed volume; *qSW*—seed width; *qSDm*—stem diameter; *qTD*—trichome density; and *qTL*—trunk length.

Of the 1325 SNP markers that showed >95% call rate across all 222 samples (88% of the 1504 total interrogated SNPs), 455 polymorphic markers are used to generate a genetic map (Figure 5). With a total length of 291.5 cM, the map consisted of 10 linkage groups, corresponding to the 10 chromosomes of *Cannabis sativa* (Table S9). The physical map generated from the same set of markers was 822.40 Mb long (Figure S8, Table S10). Chromosome 1 contained the highest number of markers (61 SNPs) while chromosome 9 had the lowest (28 SNPs). The average spacing of markers in the genetic map in each chromosome ranged from 0.4 cM to 1.4 cM, while the average gap in the physical map ranged from 1.28 Mb to 2.27 Mb (Tables S9 and S10). Chromosome X had the highest maximum space between any two markers in both the genetic map (13.7 cM) and physical map (15.46 Mb). In the genetic map, chromosomes 1 and 5 had the lowest maximum space of 5.3 cM. On the other hand, chromosome 4 had the lowest maximum gap in the physical map of 4.17 Mb.

Using the genetic map, quantitative trait loci (QTLs) were mapped for all target traits. A total of 53 QTLs with LODs exceeding the 1000 permutation test at 5% significance level were considered significant (Table S11). Among them, 25 QTLs for 14 different traits have percent variance explained (PVE) of more than 10% (Figure 5, Table 2). QTLs for seed traits were found to be in chromosomes 1, 3, 4, and 5. PVE reached as high as 48.77%, which was for seed density (*qSD3*). *qSD3* overlapped with a QTL for hundred seed weight, *qHSW3*, which had a PVE of 26.59%. *qSL3* was also found in that same region but has a wider QTL coverage (16.33 cM). The QTLs for seed length and seed volume with the highest PVEs were found in chromosome 1, *qSL1* and *qSV1,* with PVEs of 34.44% and 33.24%. There was a slight overlap between *qSL1* and *qSV1* with *qHSW1*. Chromosome 4 had multiple seed QTLs, i.e., *qSD4*, *qSV4*, and *qSW4*, with *qSW4* having the smallest region (2.6 cM) and highest PVE of 31.46%. In chromosome 5, only *qHSW5.1* had a PVE of greater than 10%, even though it overlapped with *qSL5*, *qSV5*, and *qSW5* (Table 2 and Table S11).

Plant agro-morphological traits are largely colocalized on chromosomes 2 and 9. On chromosome 2, six out of nine QTLs had PVEs of more than 10%. These QTLs were for internode count (*qIC2*, 23.60% PVE), longest branch (*qLB2*, 39.20% PVE), plant height (*qPH2*, 22.04% PVE), plant width (*qPW2*, 25.33% PVE), stem diameter (*qSDm2*, 38.12% PVE), and trichome density (*qTD2*, 10.38%). Plant height (*qPH9*) had the highest LOD of all QTLs at 61.07. It spanned only 1 cM, had a PVE of 46.99% and overlapped with *qAIL9*, *qLB9*, *qPW9*, and *qTL9*.

### 2.7. Marker-Trait Associations

**Figure 6:**
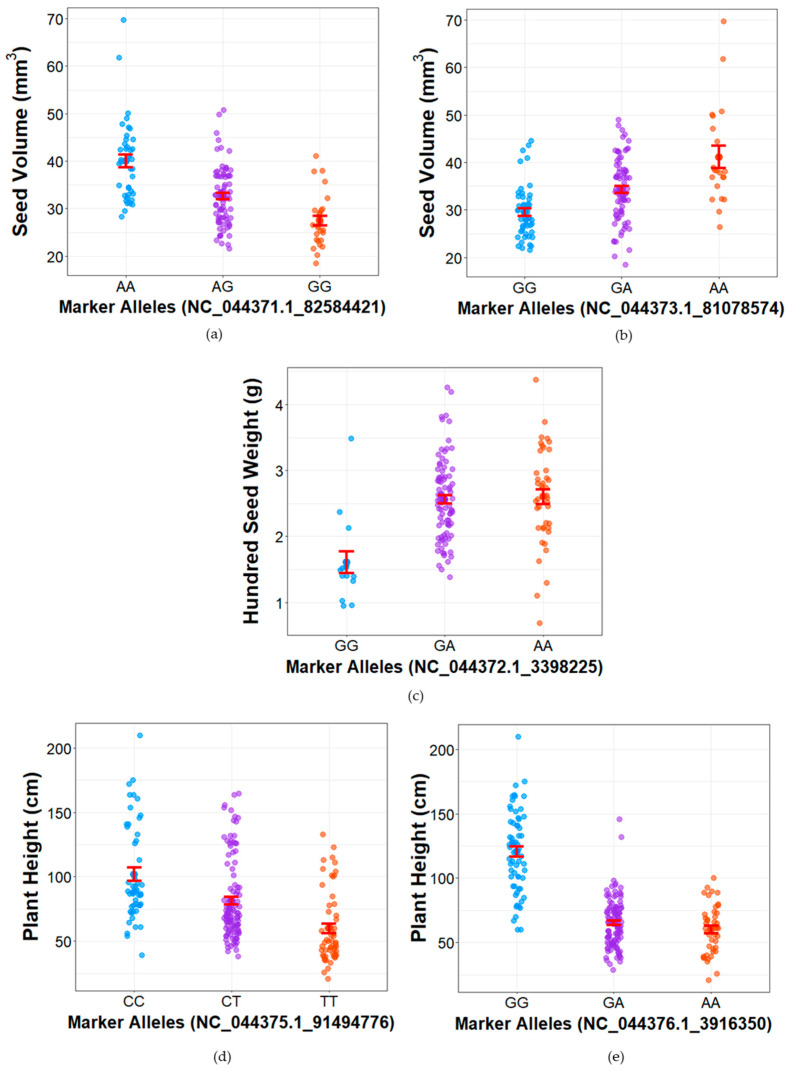
Phenotypic variation among genotypic classes at peak LOD markers in major QTLs. Phenotypes observed in the F_2_ population per allele group for peak LOD markers of seed volume on (**a**) chromosome 1 and (**b**) chromosome 4, hundred seed weight on (**c**) chromosome 3, and plant height on (**d**) chromosome 2 and (**e**) chromosome 9, with bars indicating the mean +/− standard error.

Several colocalized QTLs share common peak markers, which are associated with the observed F_2_ phenotypes (Figure 5, Table S12). NC_044371.1_82584421 on chromosome 1 is the peak marker for seed volume (Figure 6a). IPK_CAN_57 had the GG allele for this marker, which was associated with lower seed volume (27.44 ± 1.04 mm^3^) across the F_2_ population, while SI-1 had the heterozygous (AG) allele, carriers of which had a mean seed volume of 32.61 ± 0.69 mm^3^ (Table S12). Carriers of the homozygous AA allele had a significantly higher average seed volume of 40.07 ± 1.34 mm^3^. For the same marker, seed length and seed width both had the lowest means (4.64 ± 0.06 and 3.81 ± 0.05 mm) for the GG allele and the highest for the AA allele (5.29 ± 0.05 and 4.33 ± 0.05 mm) (Table S12).

For NC_044373.1_81078574, a peak marker for seed volume on chromosome 4 (Figure 6b), carriers of the AA allele had a mean of 41.14 ± 2.32 mm^3^, which was significantly different from the means of GA allele (34.31 ± 0.79 mm^3^) and GG allele (29.51 ± 0.79 mm^3^). The parent SI-1 had the AA allele while IPK_CAN_57 had the GA allele for this marker (Table S10). NC_044373.1_81078574 was also a peak marker for seed length (*qSL4*), seed width (*qSW4*), and seed density (*qSD4*) (Table S12). Seed length and seed width followed the same trend of allele-phenotype grouping, wherein the AA alleles were associated with the lowest means (4.84 ± 0.05 mm and 3.87 ± 0.03 mm), GA were intermediate (5.02 ± 0.05 mm and 4.10 ± 0.03 mm), and GG had the highest (5.11 ± 0.08 mm and 4.45 ± 0.09 mm), and all groups were significantly different from one another.

Hundred-seed weight (*qHSW3*) shows strong marker trait associations with NC_044372.1_3398225 (Figure 6c) and is further associated with seed density (Table S12). SI-1 had AA alleles for this marker while IPK_CAN_57 had a GA allele (Table S12). Hundred-seed weights for carriers of the GA and AA were similar (2.56 ± 0.06 and 2.60 ± 0.11 g, respectively), which was also observed for seed density (0.80 ± 0.01 and 0.79 ± 0.03 mg/mm^3^, respectively). Carriers of the GG allele, on the other hand, had significantly lower hundred-seed weights and seed densities at 1.61 ± 0.17 g and 0.41 ± 0.04 mg/mm^3^.

Plant height is strongly associated with NC_044375.1_91494776 on chromosome 2 (Figure 6d) and NC_044376.1_3916350 in chromosome 9 (Figure 6e). The CC allele of NC_044375.1_91494776 was associated with a tall phenotype (103.94 ± 5.14 cm) while TT was associated with a short phenotype (74.56 ± 5.30 cm), which was also the allele call for IPK_CAN_57 (Table S12). The CT allele was intermediate at 84.31 ± 3.17 cm, which was the allele for SI-1. A similar pattern was observed for NC_044376.1_3916350, where the GG allele correlated with a tall phenotype (121.48 ± 3.81 cm) and the AA allele with a short phenotype (66.42 ± 3.05 cm). The GA allele was intermediate to the other groups at 70.40 ± 1.95 cm, and was the allele called for SI-1. IPK_CAN_57, on the other hand, had the AA allele.

NC_044375.1_91494776 was also the peak marker for several agro-morphological traits, i.e., seed diameter, longest branch, plant width, trichome density, and internode count (Table S12). For stem diameter, longest branch, and plant width, phenotype values were significantly different from each allele group (CC, CT, and TT) (Table S12). Trichome density values for CC and CT alleles were not significantly different (3.02 ± 0.14 and 2.74 ± 0.09) but were different for values of the TT alleles (2.39 ± 0.14). NC_044376.1_3916350 was also associated with average internode length, longest branch, plant width, and trunk length (Table S12), showing the same allele pattern as for plant height, with high phenotype values observed for samples with the GG allele, intermediate with the GA allele, and the lowest with the AA allele.

### 2.8. Candidate Gene Selections

Several putative candidate genes for seed trait QTLs and agro-morphology QTLs located in the QTL confidence interval regions (Table 3 and Table S13) are proposed based on annotation and homology to validated trait contributing genes. Most of these were also in the closest proximity to the peak LOD marker.

For seed size QTL on chromosome 1 (*qSL1*, *qSW1* and *qSV1*), LOC115706108, a close homologue of *CESA9*, and LOC115706114, a putative orthologue of the transcriptional activator *DME*, were found within less than 100 kb of the peak LOD marker NC_044371.1_82584421. Both *CESA9* and *DME* were previously characterized to influence seed size in Arabidopsis [31,32].

LOC115714141, a cyclin-dependent kinase inhibitor with high similarity to *KRP7,* was found in proximity to NC_044373.1_81078574, the peak LOD marker for seed size QTL on chromosome 4 (*qSW4*, *qSV4,* and *qSD4*). Downregulation of *ICK/KRP* genes in Arabidopsis, including *KRP7*, resulted in bigger organs and seeds [33].

Seed weight and seed density peak LOD marker in chromosome 3 (*qHSW3* and *qSD3*) were within 1 kb of LOC115710302, a homologue of Arabidopsis *AVT1J*—an amino acid transporter [34,35] and within 100 kb of LOC115711273, a homologue of *ASML2* involved in sugar signalling in developing seeds [36,39].

For agro-morphological traits, LOC115718917 was located 47.9 kb of NC_044375.1_91494776, the peak LOD marker for the QTL cluster on chromosome 2. LOC115718917 is a homologue of *HERK1* in Arabidopsis [37]. On chromosome 9, NC_044376.1_3916350 peak marker for *qPH9*, *qAIL9*, *qLB9*, *qPW9*, and *qTL9* was located approximately 0.5 Mb from LOC115723423, a homolog of *GA2OX6*, which is involved in the regulation of gibberellins in Arabidopsis [38].

## 3. Discussion

Seed size is a key trait of the domestication syndrome. Cultivated crops, modern high-yielding cultivars in particular, tend to have much larger seed/grain than their respective wild type progenitors or landraces [40]. For most seed-based crops such as oilseeds and pulses, these increases in seed size have largely been fixed, though stark differences may remain if there are different end uses (i.e., seed and vegetable type soy (*Glycine max*) [41] and Indian mustard (*Brassica juncea*) [42]). In our hempseed diversity collection, we found a large variation with respect to seed weight and size (Figure 2a, Table S1) with an over eight-fold difference between the minimum and maximum hundred-seed weight. Likely sources of this variation include the multipurpose nature of hemp, with seed size mostly irrelevant in varieties intended for fibre or medicinal use, as well as the inclusion of non-commercial landraces in our diversity collection. In addition, targeted selection for hemp seed size simply has not happened to the same extent as in other seed crops due to the complex history of hemp [15]. This study has taken advantage of this abundance of variation to identify QTLs and associated molecular markers for seed size (Table 2). Our choice of parents reflects the sources of variation mentioned above, with SI-1 being a Chinese variety promoted for both seed and fibre use [43], while the small seed size and compact nature of the landrace IPK_CAN_57 (Figure 2c), combined with high trichome density and high CBD contents [44], point towards a medicinal use.

Maternal influence on seed size, a widely supported concept in seed biology, was evident when looking at the seed size of F_1_, F_1_-derived and F_2_-derived seeds, (a) the F_1_ seeds very closely resembled the seeds of the female parent IPK_CAN_57; (b) one generation later, seed size was about halfway between the original parents which again matches with the genotype of the F_1_ mother; (c) the F_2_-derived seeds showed a strong increase in variation compared to the parent seeds, which fits with the now segregating population of mothers that produced these seeds. This is in agreement with the concept that the seed coat, which differentiates from the maternal integuments, can set an upper limit to seed size [45]. Therefore, in crops such as hemp, the choice of maternal varieties should be carefully considered, especially when breeding for seed production type hemp.

To our knowledge, the only QTL study on hemp, that includes seed traits is a recent biparental QTL mapping study, which identified several QTLs for Thousand Seed Mass (TSM) [46]. While the parents were not specifically selected for differences in seed traits, their F_2_ population was highly variable in TSM, and the four TSM QTLs identified collectively explained 38.4% of the observed variation. Their most significant QTL, TSM.2, had a LOD of 17.2, explaining 18.2% of the variation. They also found that most TSM QTLs overlapped with QTL clusters for agro-morphological traits, and TSM showed a strong positive correlation with agro-morphological traits such as plant height and stem diameter. We identified five previously unknown seed weight QTLs, which collectively explained 66.5% of the existing variation (Table S11), with the largest seed weight QTL, *qHSW3*, havinga LOD of 14.5 and explaining 26.6% of the observed variation. Furthermore, our findings indicated that plant size and seed size were not linked (Figure 4 and Figure 5) and, therefore, breeding for seed-type ideotypes of small unbranching stature with large seeds is feasible, the opposite of what was suggested by Woods et al. [46]. This suggests that control of seed traits is complex and is likely affected by genetic backgrounds and/or growing conditions. The study by Woods et al. [46] used a monoecious and a dioecious parent with a monoecious F_1_ selfed to create the F_2_ grown in the field. This allowed variations in genes involved in flowering time regulation [47] to be expressed while pollen available for fertilization of the F_2_ would be genetically segregating, reflecting the fact that their study did not specifically focus on seed traits. In contrast, our study used pollen from the same male parent to fertilize the all-female dioecious F_2_ population under controlled conditions, ensuring that any paternal effects on seed traits were consistent across the population. Taken together, more QTL studies focusing on seed traits are recommended, ideally including genome-wide association studies that utilize the broader genetic variation in diversity panels.

Seed traits are not only separated by correlation and linkage from agro-morphological traits, but are further separated by seed size traits and seed weight traits (Figure 4 and Figure 5). This aligned with a study in soy [41], where a mapping population generated from a large-seeded oilseed variety and a small-seeded vegetable variety indicated that different loci control different aspects of morphology and weight, potentially allowing for precise breeding for desired combinations of seed shapes and weights. In our study, seed size traits (length, width, and volume) have a high positive correlation with each other (Figure 4), which aligns with previous findings [27]. Likewise, the two seed weight traits, hundred seed weight and seed density, also showed a strong positive correlation. However, hundred seed weight only weakly correlated with seed size traits, while seed density even negatively correlated with seed size traits. Seed density is an indicator of the degree of seed filling and, as such, a marker for grain quality [48]. During seed development, seed filling accounts for 10–78% of the development period, where storage reserves such as starch, proteins, and lipids accumulate [24,49]. Simply having bigger seeds does not necessarily mean that the seeds are fully filled and are nutrient-dense, and our findings suggest that the controls for seed size are partially different from the control of seed filling, with some of the F_2_-derived seed lots having high seed volume but relatively low seed weights (Figure S5b).

These differences in correlation are further reflected in the seed QTL clusters of this study. While there was overlap for seed size and hundred-seed weight on chromosome 1 and overlap for seed size and density on chromosomes 3 and 4, it is either size or weight that showed the highest LOD and PVE values for each of the seed QTL (Figure 5, Table 3). The chromosome 1 cluster affected seed size much more than weight. While *qSL1*, *qSW1* and *qSV1* had LODs of above 20 and explained more than 30% of their respective variation, *qHSW1* had a LOD of only 7.88 and a PVE of 13.3%, and seed density did not feature at all. Lines homozygous for the favourable allele of the peak marker are consistently bigger in terms of seed length and width than lines carrying the unfavourable allele, even reaching averages that are 1.5 times larger in seed volume (Figure 5, Table S12). The most promising candidate genes in this region are likely orthologues to *CESA9* and *DME*. In Arabidopsis, mutations in certain cellulose synthase (*CESA*) genes, including *cesa9*, significantly reduce seed size by affecting cell size in the seed coat during development [31]. Reduced cellulose synthesis in *cesa9* leads to smaller, less uniform epidermal cells, contributing to a smaller overall seed size. It is thus likely that aberrations in *CESA9* function or expression explain the observed differences in seed size associated with the peak marker of this QTL cluster. Alternatively, differences in function or expression of *DME*, a DNA demethylase [32,50], could be responsible. In soybeans, *GmDMEa* expression negatively correlates with seed size by epigenetically activating genes related to abscisic acid (ABA) and reducing *GmDMEa* expression correspondingly results in larger seeds [50].

The chromosome 3 cluster, on the other hand, had stronger control over seed weight and density than over size. While *qHSW3* explained more than a quarter (26.6%) of observed seed weight variation and *qSD3* accounted for nearly half of the variation in seed density (48.8%), seed volume and seed width had PVE of less than 10% while seed length had 11% (Table 3 and Table S11). LOC115711273, a likely orthologue to *ASML2*, was a high-priority candidate of this region. *ASML2* encodes a CCT domain protein that acts as a transcriptional activator for a subset of sugar-inducible genes [39]. In Arabidopsis, *ASML2* activates a similar set of genes as *ASML1/WRINKLED1*, which is a key transcription factor to control oil biosynthesis in oilseeds [39]. The *wrinkled1* mutation causes defective accumulation of seed storage oil, which is associated with reduced seed weight [51,52]. Consequently, LOC115711273 could be involved in the activation of sugar-responsive genes and the downstream control of carbon flow from sucrose import to oil accumulation in developing hempseeds. Alternatively, LOC115710302, homologous to the Arabidopsis amino acid transporter *AVT1J*, could be responsible. Amino acid transporters have been implicated in seed filling in a number of studies [34,35,53]. However, while *AVT1J* seems to be expressed in developing siliques, it has not been functionally characterized and potential roles in seed filling remain speculative. Aberrations in LOC115711273 (for oil content) or LOC115710302 (for protein content) function are suggested to affect seed filling rather than size and hence explain the difference in weight and density associated with *qHSW3* and *qSD3*, without having a strong effect on size.

The chromosome 4 seed QTL cluster was again more pronounced in controlling size rather than weight. It explained about one quarter of the variation in seed volume (PVE for *qSV4* 23.3%), which seemed to be driven largely by seed width (overlapping *qSW4* PVE of 31.5%), while the overlapping *qSL4* only had a PVE of 8.5% (Table 3 and Table S11). Lines carrying the AA allele for peak marker NC_044373.1_81078574 were more than 25% larger by volume than GG allele carriers, and the intermediate phenotype of GA lines suggested incomplete dominance (Table S12). LOC115714141, a likely orthologue of the Arabidopsis *CDKI7,* was constituted as a strong candidate gene for this chromosome 4 seed QTL cluster. *CDKIs*, including *CDKI7*, have been implicated in seed size control in a number of studies. In Arabidopsis, where *CDKIs* seem to have redundant functions, downregulation of multiple *CDKIs* resulted in larger seeds in a dose-dependent fashion, presumably through release of *CDK* inhibition and downstream stimulation of cell proliferation [33]. Dose dependency fits with the observed incomplete dominance of the linked NC_044373.1_81078574 marker (Figure 6b). In rice, two *CDKIs* were observed to be mainly expressed in developing seeds and were positively responsive to abscisic acid and brassinosteroid signals [54]. Overexpression as well as knock-out of these *CDKIs* negatively affected grain filling and corresponding seed size, suggesting roles in control of the steady-state of cell proliferation and expansion in developing seeds. In cotton, the *CDKI GhKRP6* was also induced by brassinosteroid, and downregulation negatively affected cell expansion in seeds, leading to thinner and shorter seeds. For example, in Arabidopsis, *CDKI* downregulation resulted in *CDK* upregulation.

Among the agro-morphological traits, plant height seems most relevant in the context of developing highly productive hemp seed cultivars. Reduced and uniform height is paramount for modern grain and seed crops, and reduction in plant height at increased yields has been a key driver of the green revolution and associated improvements in harvest index and mechanization [55,56,57]. Furthermore, plant height positively correlates with other agro-morphological traits of agronomic relevance (Figure 4), and both plant height QTLs further clustered with these other traits in chromosomes 2 and 9 (Figure 5). Chromosome 2 featured a multi-QTL cluster for agro-morphological traits, including height (Figure 5), most of which are highly correlated (Figure 4). A genome-wide association study (GWAS) of Iranian cultivars showed a similar colocalization of QTL for plant height and number of nodes on chromosome 2 [58]. Other studies, including the study by Woods et al. [46] and a GWAS of Canadian accessions [59], also detected QTL clusters for agro-morphological traits on chromosome 2, suggesting that these QTLs are robust across a range of hemp germplasm (Table S14). Interestingly, *qTD2* (LOD = 5.28, PVE = 10.38%) for trichome density also colocalized with the agro-morphological traits despite showing little to no correlation with plant height (Figure 4 and Figure 5), implying a genetic mechanism spanning cell expansion, proliferation and differentiation. In Arabidopsis, gibberellin, a known phytohormone that controls plant height [60], also influences trichome initiation and morphogenesis [61]. The strongest candidate in the flanking regions of the peak LOD marker was LOC115718917, coding for a putative orthologue of the receptor-like protein kinase *HERK1* (Table 3). *HERK1* was shown to be involved in cell elongation, with mutants showing dwarfed phenotypes [37]. *HERK1* was further responsive to brassinosteroid and gibberellin signalling [37], which could potentially link plant size and trichome density phenotypes.

Chromosome 9 also contains a multi-QTL cluster for agro-morphological traits (Table 3, Figure 5). It was responsible for nearly half of the variation for plant height (*qPH9* PVE 47%) and internode length (*qIL9* PVE 45.5%), and individuals homozygous for the AA allele of the peak marker were nearly twice as tall as heterozygotes or GG carriers. This strong control of plant height, again, suggests implications of gibberellin pathways, which have been broadly utilized to reduce height across various crop species [60]. LOC115723423, a gibberellin 2-beta-dioxygenase 2, is a homologue of Arabidopsis *GA2OX6*, which is a gibberellin oxidase (Table 3). Previous studies show that *GA2OX6* have activities that regulate gibberellins (GA), particularly GA_1_, GA_4_, and GA_19_, by inactivating them [38]. These GAs are all major players in the gibberellin pathway, and their oxidation limits the available GA, leading to suppression of elongation of the main stem and side shoots, and dwarf phenotypes [38,62].

## 4. Materials and Methods

### 4.1. Plant Materials and Crosses

Parents for the biparental mapping population were selected from a collection of 84 accessions available in the Southern Cross University hemp diversity collection (Table S1). Genotype data of the collection were obtained from a previous study [63]. A neighbour-joining phylogenetic tree of the collection was generated from TASSEL [64] using a modified Euclidean distance model. Selection of the parents is described in the results. A female IPK_CAN_57 plant was pollinated with a male SI-1 plant to produce F_1_ seeds (Figure S3). Female F_1_s were treated with silver thiosulfate (STS) to induce the production of male flowers [65]. The treatment allowed the plants to self-pollinate and produced F_1_-derived seeds, which, when planted, generated the all-female F_2_ population. Plants were grown and all materials maintained under NSW Low-THC Industrial Hemp Licence no. 52204 or NSW Health Authority A-202304-435/A-202503-1062 following all relevant regulations and legislative requirements.

### 4.2. Cultivation and F_2_ Pollination

An initial batch of 280 F_1_-derived seeds was germinated, and 104 seedlings were transplanted to hiko trays two days later. A second batch of 160 F_1_-derived seeds was germinated 2 weeks after the first, and 118 were transplanted to hiko trays as well. For all cultivations, the potting mix used was a combination of cocopeat (40%), engineered wood fibre (40%), and perlite—medium P400 (20%) with fertilizers and additives composed of 1:3 lime: mudgee dolomite mix, Go Grow Trace Element blend, natural gypsum, inoculated zeolite, and Osmocote Exact Standard. The seedlings were placed in a cultivation tent with long photoperiod (18/6 h light/dark) conditions for a 4-week vegetative phase. The plants were then transferred to 2 L pots and were placed on benches with a capillary watering setup that gives the bottom of the pots access to water. While on the benches, the plants were exposed to short photoperiod (11/13 h light/dark) conditions to induce flowering. Approximately 3 weeks after initiation of flowering, the plants were treated preventively with biocontrol agents *Neoseiulus californicus*, *Dalotia coriaria* and *Hypoaspis miles* to target pests such as aphids, spider mites, and fungus gnats. A total of 222 F_2_ plants reached maturity.

Pollen was harvested beforehand from a male SI-1 plant. Metal pans were placed inside growth chambers to collect the pollen when shaking the plant, which was then freeze-dried and stored at −20 °C until use. When the flowers of the feminized F_2_ population reached maturity, the plants were pollinated individually with the harvested SI-1 pollen using a puffer in a staggered manner as pistils matured and stigmas became receptive. Phenotyping and harvesting were also performed in a staggered manner, 11 to 13 weeks after pollination, as seeds finished ripening. All 222 plants were harvested and threshed, and 203 produced seeds, which were phenotyped as the F_2_-derived seeds.

### 4.3. Phenotyping

A set of 100 seeds for each accession in the SCU hemp diversity collection was weighed for hundred seed weight. For accessions with less than 100 seeds, the whole seed lot was weighed, and the hundred seed weight was computed by dividing the total weight by the total number of seeds multiplied by 100. The same sets of seeds were then scanned using an Epson flatbed scanner at 300 dots per inch (dpi). Images generated were analyzed through GrainScan [66], measuring the seed length, seed width, and seed area. For each line, the mean seed length and width were computed.

Additionally, manual measurements of seed weight, length, width, and thickness (Figure 1b) were also measured for selected seed groups. For the seeds of the parents SI-1 and IPK_CAN_57, the F_1_, and the F_1_-derived, a representative set of 25 seeds were used. As for the F_2_-derived seeds, 51 seed lots were selected from the 203 available and 10 seeds per seed lot were manually measured, totalling 510 seeds. To compare the values for F_2_-derived seeds with the other seed groups, the measurements for the 10 seeds were averaged to represent the seed lot, resulting in a sample count of 51. Seed volume was calculated using the formula $V=k_{0}\cdot Seed\;Length\;\cdot Seed\;Width\;\cdot Seed\;Thickness$, where $k_{0}=0.455$ as previously determined [30].

The phenotype data of SI-1, IPK_CAN_57, and the F_2_-derived seeds were then used to determine the relationship of seed thickness to seed length and seed width. Seed width and seed thickness were fitted to a simple linear regression model. The model was used in predicting seed thickness for the rest of the 203 F_2_-derived seed lots.

All of the 203 F_2_-derived seed lots were phenotyped through the same method as the SCU hemp diversity collection. With seed length and width available for these seed lots, seed thickness was then predicted using the linear model. Finally, the seed volume was calculated, and the seed density was calculated as: $Seed\;density=\frac{Average\;mass\;per\;seed}{Average\;volume\;per\;seed}$. Seed lots with fewer than 20 seeds were excluded before further analyses were performed, bringing the total to 147 seed lots.

For agronomic traits, all observations were performed right before harvesting of each individual F_2_ plant. These include plant height, plant width, trunk length, internode count, length of longest branch, stem diameter, and pith diameter (Table 4). Aside from these, variations were observed in the trichome density and compactness of the inflorescence of the population, and thus, a system was created to score the F_2_ individuals for these traits as well (Figures S9 and S10).

### 4.4. Statistical Analyses

Kruskal-Wallis test [67] and Dunn’s test [68] were performed for group comparisons. Correlations were determined through Pearson’s correlation coefficient [69]. All statistical analyses and plots were generated in R (version 4.4.0) [70] through Rstudio (version 2023.12.0+369, Ocean Storm) [71] using R/FSA [72], R/tidyverse [73], R/ggplot2 [74], R/corrplot [75], and R/patchwork [76] packages.

### 4.5. Genotyping

Leaf sampling was performed six weeks after transplanting, and samples collected were stored in −20 degrees Celsius freezers. DNA extraction was performed using commercial kits (DNeasy Plant Mini Kits, Qiagen USA, Valencia, CA, USA). Quality and quantity of the extracted samples were checked using gel electrophoresis and a NanoDrop 2000 spectrophotometer (Thermo Scientific, Wilmington, DE, USA). The samples were then sent to Diversity Arrays Technology (DArT), Canberra, Australia, for high-throughput genotyping using the HASCH panel [63].

### 4.6. Genetic Map and QTL Mapping

All of the following analyses were performed using R/qtl [77]. The raw genotype data of the F_2_ population from the HASCH panel were converted to R/qtl input file. The data was filtered to remove markers with low call rates (<95%), duplicated markers, and those exhibiting significant segregation distortion at the 5% level.

A linkage map was constructed using the retained markers. Markers were assigned to linkage groups based on a maximum recombination fraction of 0.35 and a minimum LOD score of six to ensure strong linkage between markers [77]. Once the linkage groups were established, the recombination fractions were converted to centimorgans (cM) using the Kosambi mapping function. Lastly, the resulting genetic map was re-estimated through the Lander-Green algorithm [78]. This constructed map was subsequently used in QTL mapping, along with log_10_-transformed phenotype data, for the seed and agro-morphological traits observed in this study.

For QTL mapping, genotype probabilities were calculated at 1 cM intervals, and a single-QTL genome scan was performed through the Haley-Knott regression method. To check for interacting loci and to separate linked QTLs, a two-dimensional scan was also performed using the same method but with a density of 2 cM. Significance thresholds were determined using permutation testing (n = 1000). To further analyze the single- and two-QTL genome scans, a multiple-QTL analysis was performed, again using the Haley-Knott regression method. The resulting genotype probabilities were used in a stepwise QTL selection approach and then further refined to improve QTL position accuracy. Confidence intervals for QTL positions were defined using 95% Bayesian credible intervals. From here, the QTL model was fitted through the Haley-Knott regression method, estimating the final LOD scores and phenotypic variance explained (PVE) by identified QTLs. QTLs exceeding the permutation-derived genome-wide threshold were considered significant, and PVE scores of ≥10% were considered major QTLs [79]. Graphical illustration of the genetic and QTL map was performed through MapChart [80]. Given the positions of the markers, a physical map was also generated through MapChart.

### 4.7. Identification of Potential Candidate Genes

Predicted peptide sequences of annotated genes of the Cannabis genome (CBDRx) located within the QTL confidence interval of the peak LOD marker per trait were downloaded from the International Cannabis Genomics Research Consortium (ICGRC) (https://icgrc.info, 23 August 2025 [81]). These sequences were aligned to the best BLASTP matches within the organism Phytozome 14 (https://phytozome-next.jgi.doe.gov, 23 August 2025 [82]) database, using Arabidopsis (Araport11) as a reference organism, with a cut-off of ≥35% alignment length and ≥60% sequence identity, retaining only the top hit for each query. Arabidopsis gene annotation from TAIR (https://www.arabidopsis.org, 23 August 2025 [83]) was used to functionally characterize each candidate gene in each QTL.

## 5. Conclusions

Modern plant breeding approaches increasingly utilize QTLs and associated molecular markers to reduce time and cost requirements for the improvement of plant architecture and seed/grain quality to adapt crops to specific environments and end uses [84,85,86,87,88]. Although hemp has been cultivated for thousands of years, its advancement as a modern crop is still in its infancy [15], having been largely ignored in the green revolution for regulatory reasons. While genetic and genomic resources for hemp have significantly improved over the last decade paving the way for modern pre-breeding and breeding [89] including marker-assisted selections, the availability of robust QTLs for key traits for hemp improvement remains limited. Available QTLs focus on traits involved in cannabinoid production, fibre quality, sex expression, flowering, and disease resistance [46,58,59,90,91,92]. In this study, an additional 53 novel QTLs for 15 different traits are described with a particular focus on seed traits, which provide an avenue for improving hemp as an oilseed and seed protein crop. Candidate genes, selected based on proximity to peak LOD markers for the most promising QTLs and their homology to known Arabidopsis genes involved in seed traits and agro-morphological phenotypes, are suggested. Although highly speculative, they can serve as a starting point for further studies. Furthermore, the absence of positive correlations between seed traits and plant agro-morphological traits in this study further suggests that an oilseed ideotype—short in stature, low in branching, yet producing large, dense seeds—is attainable for hemp, an emerging crop that is still full of untapped potential.

## Figures and Tables

**Table 1:** Summary of all the phenotypes measured in the F_2_ population from the cross between SI-1 and IPK_CAN_57.

Trait	Abbreviation	Unit of Measurement	Number of Samples	Min	Max	Mean	SD ^1^	CV ^2^ (%)
Hundred Seed Weight	HSW	g	147	0.7	4.4	2.5	0.7	28.6
Seed Length	SL	mm	147	3.9	6.0	5.0	0.4	7.7
Seed Width	SW	mm	147	3.4	5.4	4.1	0.3	8.4
Seed Volume	SV	mm^3^	147	18.1	66.4	32.5	7.6	23.3
Seed Density	SD	mg/mm^3^	147	0.3	1.1	0.8	0.2	26.3
Plant Height	PH	cm	222	21.0	210.0	80.5	35.1	43.6
Trunk Length	TL	cm	222	1.0	31.0	9.8	5.8	59.1
Internode Count	IC	integer	222	5.0	23.0	10.8	2.9	26.7
Average Internode Length	AIL	cm	222	2.3	15.1	6.5	2.5	37.8
Plant Width	PW	cm	222	5.0	89.0	28.7	12.5	43.6
Longest Branch	LB	cm	222	4.0	90.0	27.5	15.0	54.4
Stem Diameter	SDm	mm	222	3.0	13.4	8.3	1.9	23.0
Pith Cavity Diameter	PDm	mm	222	0.0	6.0	2.4	1.2	48.1
Trichome Density	TD	score	222	0.0	5.0	1.9	1.1	55.3
Inflorescence Compactness	C	score	222	1.0	5.0	2.7	0.9	32.7

^1^ SD—standard deviation; ^2^ CV—coefficient of variation.

**Table 2:** Quantitative trait loci (QTLs) found for the traits observed in this study with LOD exceeding the 1000 permutation test threshold at 5% significance level and percent variance explained of more than10%. QTL of the largest LOD and PVE for each trait are highlighted in bold.

Trait	QTL	Chr	Peak LOD Marker	Location (cM)	CI ^1^ (cM)	LOD ^2^	PVE ^3^ (%)
Average Internode Length	***qAIL9***	**9**	**NC_044376.1_7017434**	**6.00**	**5.00–7.00**	**33.95**	**45.53**
Hundred-Seed Weight	***qHSW3***	**3**	**NC_044372.1_3398225**	**9.00**	**7.00–10.03**	**14.15**	**26.59**
	*qHSW1*	1	NC_044371.1_89081398	16.42	12.12–18.00	7.88	13.34
	*qHSW5.1*	5	NC_044374.1_364024	0.00	0.00–0.00	6.53	10.82
Internode Count	***qIC2***	**2**	**NC_044375.1_91494776**	**6.00**	**1.00–15.00**	**14.38**	**23.60**
Longest Branch	***qLB2***	**2**	**NC_044375.1_74061285**	**2.49**	**1.59–9.00**	**28.06**	**39.20**
	*qLB9*	9	NC_044376.1_3916350	6.00	3.00–9.19	19.51	24.23
Plant Height	***qPH9***	**9**	**NC_044376.1_3916350**	**6.00**	**5.00–6.00**	**61.07**	**46.99**
	*qPH2*	2	NC_044375.1_91494776	5.26	0.00–7.58	37.92	22.04
Plant Width	***qPW2***	**2**	**NC_044375.1_79822397**	**8.03**	**1.00–9.00**	**18.76**	**25.33**
	*qPW9*	9	NC_044376.1_3916350	5.00	1.00–10.09	9.93	12.17
Seed Density	***qSD3***	**3**	**NC_044372.1_3398225**	**9.00**	**8.00–10.03**	**23.65**	**48.77**
	*qSD4*	4	NC_044373.1_81078574	22.56	22.00–29.00	7.87	12.43
Seed Length	***qSL1***	**1**	**NC_044371.1_82584421**	**12.12**	**9.41–15.00**	**20.06**	**34.44**
	*qSL3*	3	NC_044372.1_4031901	11.00	7.00–23.33	7.91	11.07
Seed Volume	***qSV1***	**1**	**NC_044371.1_82584421**	**12.12**	**10.00–15.00**	**22.37**	**33.24**
	*qSV4*	4	NC_044373.1_81078574	22.56	21.00–24.60	17.15	23.28
Seed Width	***qSW4***	**4**	**NC_044373.1_81078574**	**23.00**	**22.00–24.60**	**22.51**	**31.46**
	*qSW1*	1	NC_044371.1_80937586	12.57	10.00–14.00	21.98	30.44
Stem Diameter	***qSDm2***	**2**	**NC_044375.1_91494776**	**5.26**	**2.00–8.48**	**32.36**	**38.12**
Pith Cavity Diameter	***qPDm4.2***	**4**	**NC_044373.1_19597734**	**29.18**	**29.18–29.18**	**11.89**	**19.16**
	*qPDm4.1*	4	NC_044373.1_63812876	28.00	28.00–28.51	10.73	17.08
Trichome Density	***qTD2***	**2**	**NC_044375.1_6469635**	**2.04**	**0.00–11.00**	**5.28**	**10.38**
Trunk Length	***qTL9***	**9**	**NC_044376.1_3916350**	**3.00**	**0.00–7.00**	**9.82**	**14.73**
	*qTL4*	4	NC_044373.1_46738638	26.55	23.70–29.41	7.88	11.57

^1^ CI—confidence interval; ^2^ LOD—logarithm of the odds; ^3^ PVE—percent variance explained.

**Table 3:** Top candidate genes based on CBDRx and Arabidopsis annotation found within QTL confidence intervals of peak LOD markers for QTLs identified in this study.

QTL	QTL Peak LOD Marker	Distance from Marker (kbp)	CBDRx GeneID	CBDRx Annotation	Arabidopsis AGI ^1^ Identifier	Arabidopsis Annotation ^2^	Reference
*qSL1*, *qSW1*, *qSV1*	NC_044371.1_82584421	19.9	LOC115706108	glucomannan 4-beta-mannosyltransferase 9	AT5G03760	*CESA9*	[31]
		84.2	LOC115706114	transcriptional activator DEMETER, transcript variant X3	AT5G04560	*DME*	[32]
*qSW4*, *qSV4*, *qSD4*	NC_044373.1_81078574	111.4	LOC115714141	cyclin-dependent kinase inhibitor 7	AT1G49620	*KRP7*	[33]
*qHSW3*, *qSD3*	NC_044372.1_3348225	0.8	LOC115710302	amino acid transporter AVT1J, transcript variant X1	AT5G15240	*AVT1J*	[34,35]
		97.7	LOC115711273	probable cyclin-dependent serine/threonine-protein kinase DDB_G0292550	AT3G12890	*ASML2*	[36]
*qSDm2*, *qLB2*, *qPH2*, *qPW2*, *qTD2*, *qIC2*	NC_044375.1_91494776	47.9	LOC115718917	receptor-like protein kinase HERK 1	AT3G46290	*HERK1*	[37]
*qPH9*, *qAIL9*, *qLB9*, *qPW9*, *qTL9*	NC_044376.1_3916350	486.4	LOC115723423	gibberellin 2-beta-dioxygenase 2	AT1G02400	*GA2OX6*	[38]

^1^ AGI—Arabidopsis Genomic Initiative; ^2^ Abbreviations: *CESA9—Cellulose Synthase Like A9*; *DME—Demeter*; *KRP7—Kip-Related Protein 7*; *AVT1J—Amino Acid Vacuolar Transporter 1J*; *ASML2—Activator Of Spomin::LUC2*; *HERK1—Hercules Receptor Kinase 1*; *GA2OX6—Gibberellin 2-Oxidase 6*.

**Table 4:** Agronomic traits observed in this study and how they were measured.

Variable	Unit	Measurement
Plant height	cm	Measure from the ground soil to the tip of the standing plant
Plant width	cm	Measure from the outermost tip to the tip of the standing plant
Trunk length	cm	Measure the ground soil to the first branch, excluding the node with only leaves
Internode count	integer	Count the number of all nodes, including those without branches, while excluding the compact top with alternating pattern
Average internode length	cm	Compute $\frac{Plant\;Height-Trunk\;Length}{Number\;of\;Internodes}$
Longest branch	cm	Measure the longest branch from the main stem to the tip
Stem diameter	mm	Measure at the middle of the first smooth internode
Pith cavity diameter	mm	Measure the hollow part of the pith where the stem diameter was measured
Trichome density	scale	Compare with representative plants and score from 0 to 5 (Figure S9)
Inflorescence Compactness	scale	Compare the compactness of the main bud with representative plants and score from 1 to 5 (Figure S10)

## Data Availability

The original contributions presented in this study are included in the article/Supplementary Material. Further inquiries can be directed to the corresponding author.

## Supplementary Materials

The following supporting information can be downloaded at: https://www.mdpi.com/article/10.3390/plants14243853/s1, Figure S1: Seed length and seed width of 84 available hemp cultivars in the SCU collection, highlighting the parents IPK_CAN_57 (red) and SI-1 (blue); Figure S2: Phylogenetic tree of 84 available hemp cultivars in the SCU collection, highlighting the parents IPK_CAN_57 (red) and SI-1 (blue), their F_1_ (yellow), and their F_2_ (purple) offsprings; Figure S3: Schematic diagram of the biparental cross between IPK_CAN_57 and SI-1 and where the F_1_, F_1_-derived and F_2_-derived seeds were sourced; Figure S4: Comparison of relationships of seed width and seed length to seed thickness based on measured data of SI-1 (n = 25), IPK_CAN_57 (n = 25), and F_2_-dervied seeds (n = 510) to determine prediction fitness for seed thickness; Figure S5: (a) Seed traits measured for SI-1 (n = 25), IPK_CAN_57 (n = 25), F_1_ (n = 25), F_1_-derived (n = 25), and F_2_-derived seeds (n = 51); (b) Correlation between seed weight and seed volume based on SI-1 (n = 25), IPK_CAN_57 (n = 25), F_1_ (n = 25), F_1_-derived (n = 25), and F_2_-derived seeds (n = 51); Figure S6: Distribution of seed traits—(a) hundred seed weight, (b) seed volume, (c) seed length, (d) seed width, and (e) seed density—of the F_2_-derived seeds generated from the cross between SI-1 and IPK_CAN_57 (n = 147); Figure S7: Distribution of agronomic traits—(a) plant height, (b) trunk length, (c) number of internodes, (d) average internode length, (e) plant width, (f) longest branch, (g) stem diameter, (h) pith cavity diameter, (i) inflorescence compactness, and (j) trichome density—of the F_2_ population generated from the cross between SI-1 and IPK_CAN_57 (n = 222); Figure S8: The physical positions of the 455 genetic markers used in this study; Figure S9:Trichome density scale followed in this study based on varying degrees of trichome density of six plants selected from the F_2_ population; Figure S10: Inflorescence compactness scale followed in this study based on varying degrees of inflorescence compactness of three plants selected from the F_2_ population; Table S1. (a) Hundred seed weight, seed length, seed width, and seed volume and (b) statistical summary of 84 hemp cultivars available in the SCU hemp collection; Table S2. Genotype data of the SCU hemp collection, and the F_1_ and F_2_ individuals from the cross between SI-1 and IPK_CAN_57, generated from the HASCH panel; Table S3. Measured seed length, width, and thickness of SI-1 (n = 25), IPK_CAN_57 (n = 25), and 51 (n = 10) F_2_-derived accessions used for modelling seed thickness prediction across all F_2_-derived seeds; Table S4. Model fit for linear regression of seed thickness on seed width based on parental (SI-1 and IPK_CAN_57) and F_2_ population data; Table S5. Measured seed length, width, thickness, and weight of SI-1 (n = 25), IPK_CAN_57 (n = 25), F_1_ (n = 25), F_1_-derived (n = 25), and F_2_-derived (n = 51 where each accession is the average of 10 seeds); Table S6. Means and standard error of means of seed traits of SI-1 (n = 25), IPK_CAN_57 (n = 25), F_1_ (n = 25), F_1_-derived (n = 25), and F_2_-derived (averaged values from 51 accessions) and their statistical groupings across seed groups based on Kruskal–Wallis and Dunn’s test; Table S7. All phenotype data of the F_2_ population (n = 222) from the cross between SI-1 and IPK_CAN_57; Table S8. Pearson correlation coefficients and *p*-values (in parenthesis) for each correlation between all the traits observed in this study; Table S9. Summary of the genetic map generated from the genotype data of the F_2_ population of the cross between SI-1 and IPK_CAN_57; Table S10. Summary of the physical positions of the 455 genetic markers used in this study; Table S11. All the quantitative trait loci (QTLs) found for all the traits observed in this study; Table S12. F_2_ population phenotype mean values and standard error of means for each allele group (A for reference alleles, B for alternative alleles, and H for heterozygous alleles) of selected peak LOD markers, statistically grouped across allele groups based on Kruskal–Wallis and Dunn’s test; Table S13. Putative candidate genes based on CBDRx and Arabidopsis annotation were found within QTL confidence intervals of peak LOD markers for QTLs identified in this study; Table S14. QTLs identified in this study, as compared to previously identified QTLs from different studies, and their relative positions based on CBDRx annotation.

## Abbreviations

The following abbreviations are used in this manuscript:

QTL	Quantitative Trait Loci
SNP	Single Nucleotide Polymorphism
PVE	Percent variance explained
THC	Δ9-tetrahydrocannabinol
PUFA	Polyunsaturated fatty acids
LA	Linoleic acid
ALA	Alpha-linolenic acid
CV	Coefficient of Variation
HSW	Hundred Seed Weight
SL	Seed Length
SW	Seed Width
SV	Seed Volume
SD	Seed Density
PH	Plant Height
TL	Trunk Length
IC	Internode Count
AIL	Average Internode Length
PW	Plant Width
LB	Longest Branch
SDm	Stem Diameter
PDm	Pith Cavity Diameter
TD	Trichome Density
C	Inflorescence Compactness
SD	Logarithm of the odds
CI	Confidence interval
LOD	Logarithm of the odds
*CESA9*	*Cellulose Synthase Like A9*
*DME*	*Demeter*
*KRP7*	*Kip-Related Protein 7*
*AVT1J*	*Amino Acid Vacuolar Transporter 1J*
*ASML2*	*Activator Of Spomin::LUC2*
*HERK1*	*Hercules Receptor Kinase 1*
*GA2OX6*	*Gibberellin 2-Oxidase 6*
TSM	Thousand seed mass
STS	Silver thiosulfate
