# Supplementary material for: Seed the Difference: QTL Mapping Reveals Several Major Loci for Seed Size in Cannabis sativa L
Source: Plants (Basel). 2025 Dec 17;14(24):3853. doi: 10.3390/plants14243853 (PMC12737016; doi:10.3390/plants14243853)
Supplement: Supplementary file 1 [file plants-14-03853-s001.zip › revised supplementary materials_manansala-siazon et al/20251126_supplementaryfigures.pdf]

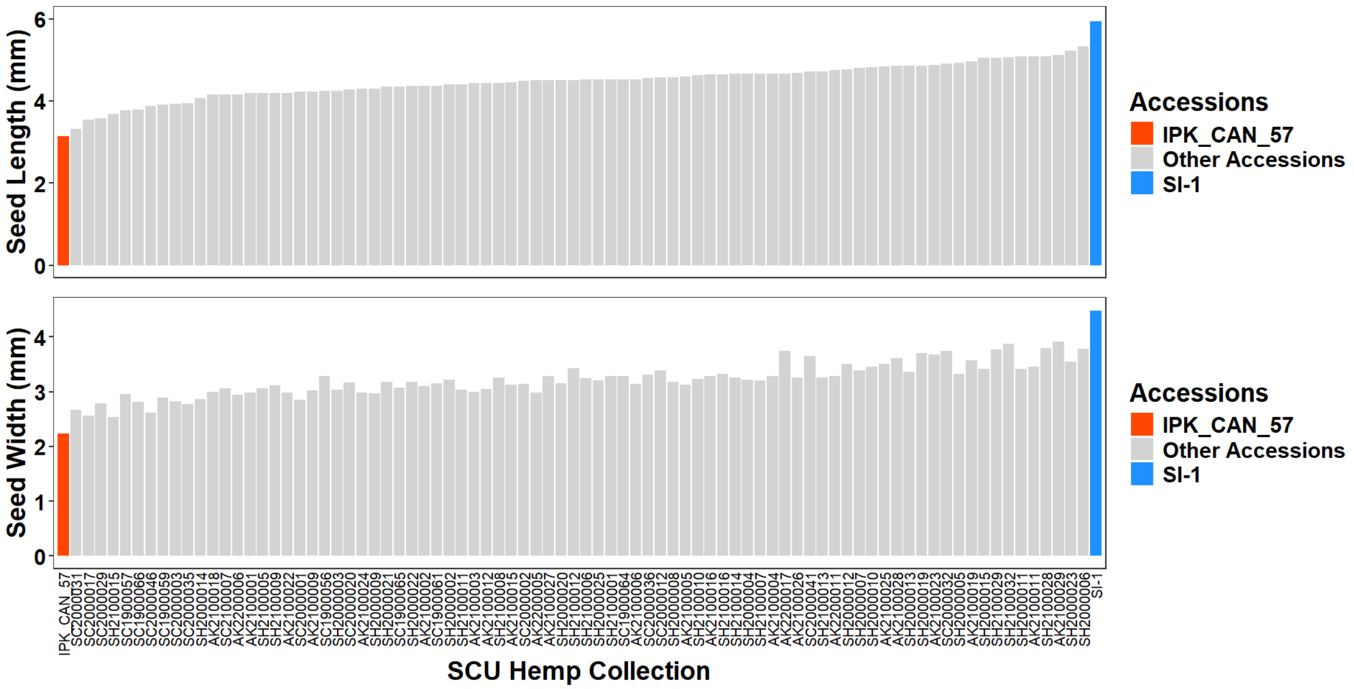

Figure S1. Seed length and seed width of 84 available hemp accessions in the SCU collection, highlighting the parents IPK_CAN_57 (red) and SI-1 (blue).

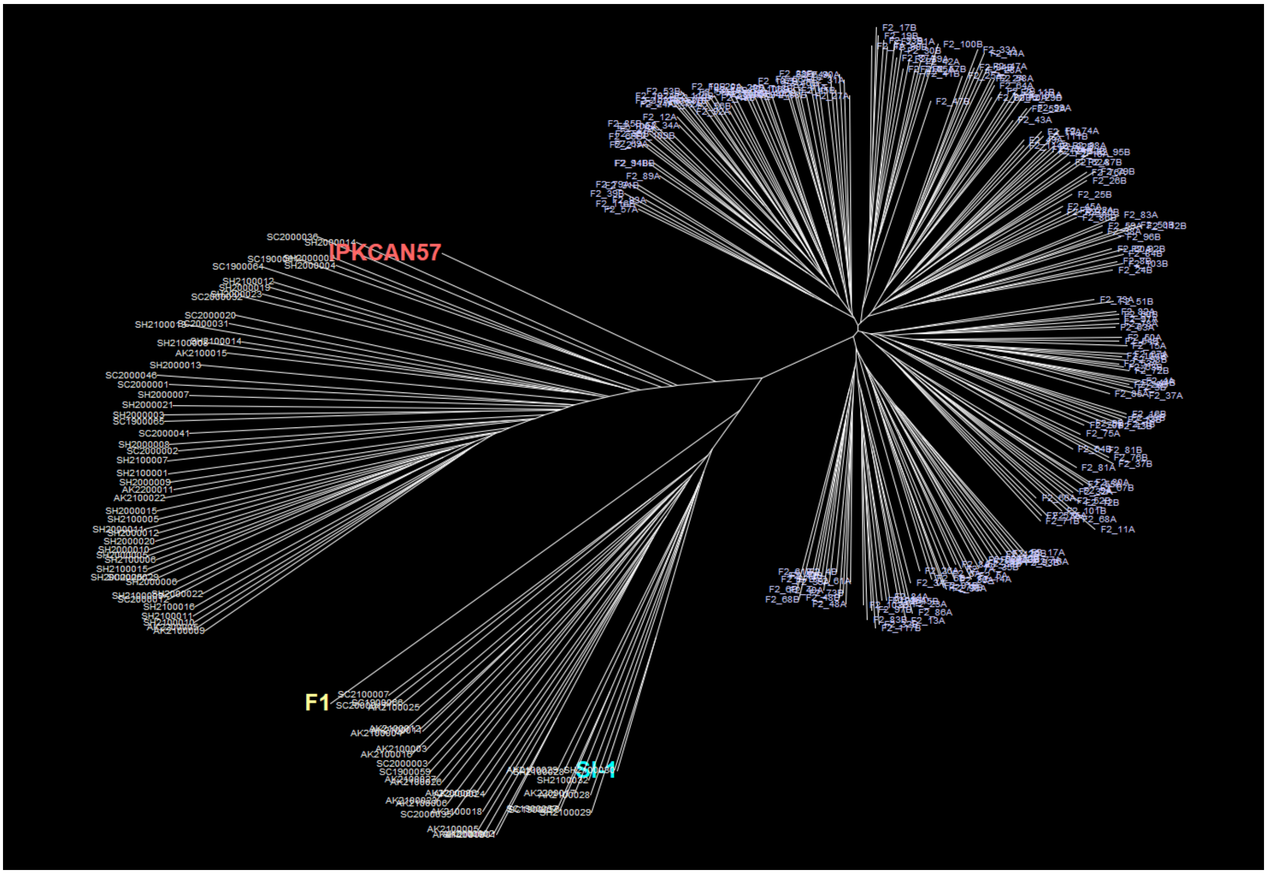

Figure S2. Phylogenetic tree of 84 available hemp accessions in the SCU collection, highlighting the parents IPK_CAN_57 (red) and SI-1 (blue), their F_1 (yellow), and their F_2 (purple) offsprings.

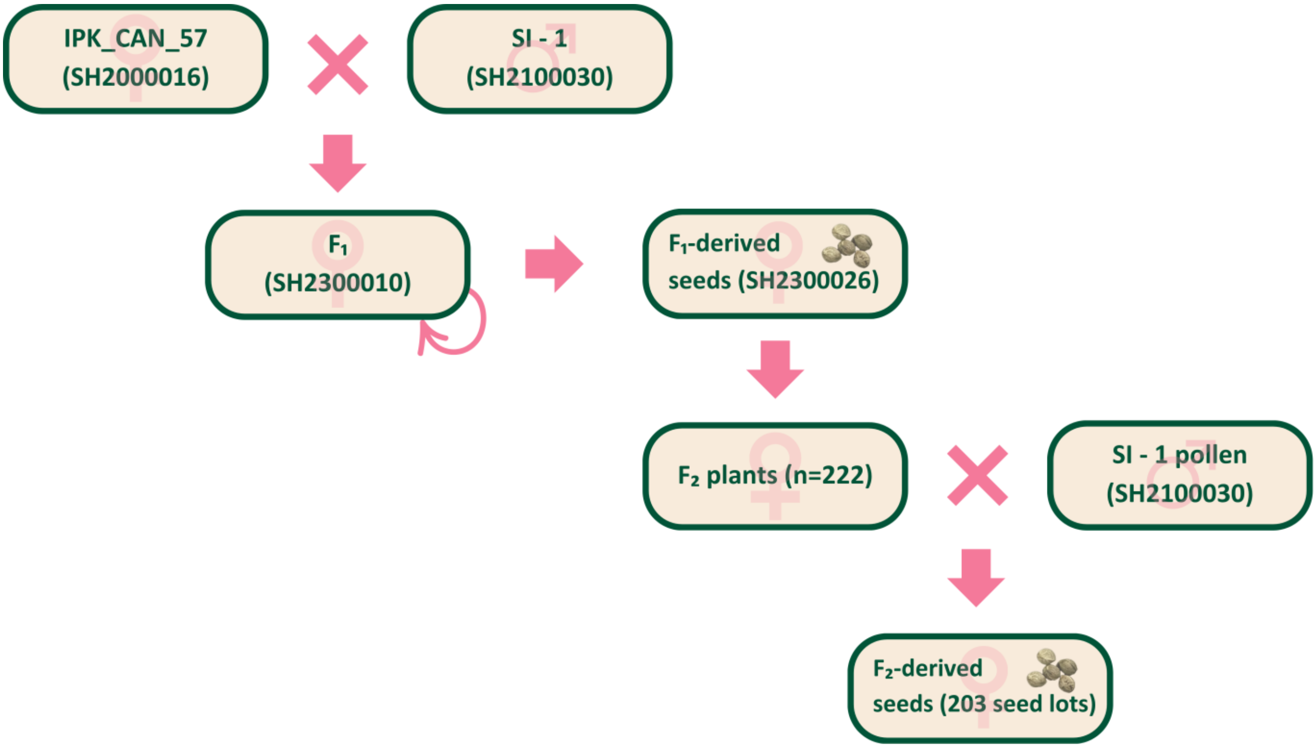

Figure S3. Schematic diagram of the biparental cross between IPK_CAN_57 and SI-1 and where the F_1, F_1-derived and F_2-derived seeds were sourced.

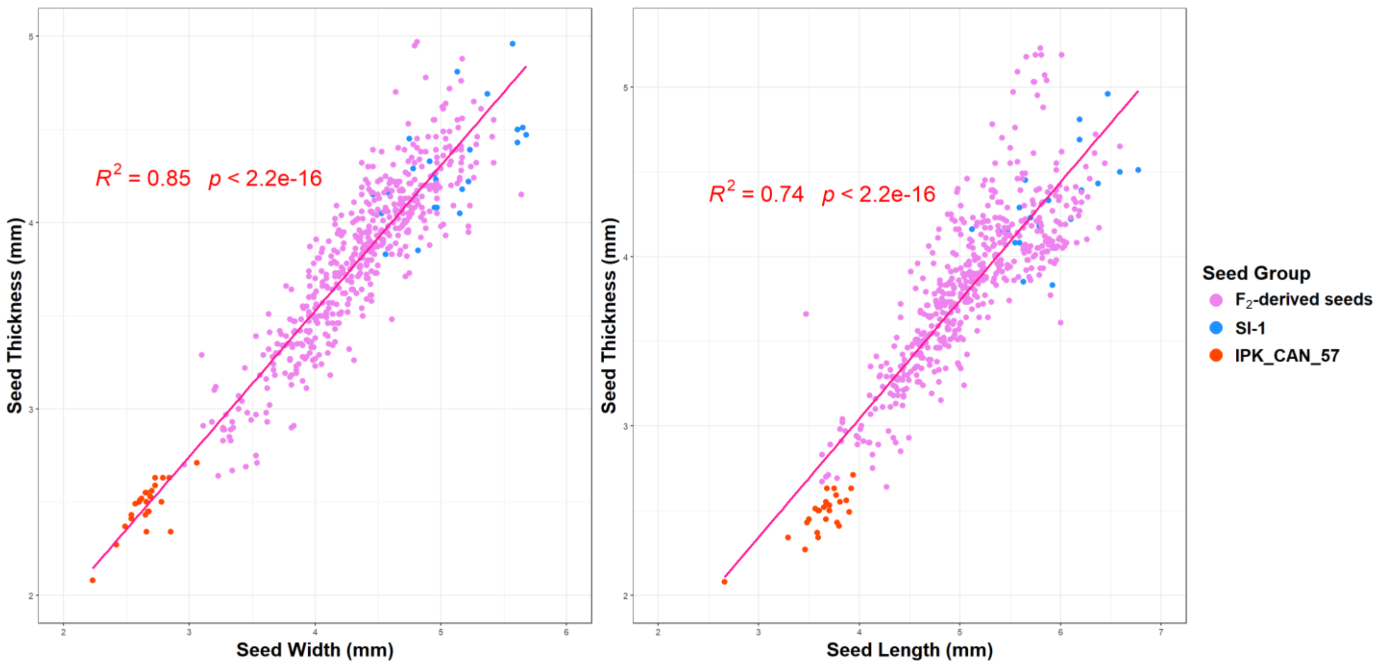

Figure S4. Comparison of relationships of seed width and seed length to seed thickness based on measured data of SI-1 (n=25), IPK_CAN_57 (n=25), and F_2-dervied seeds (n=510) to determine prediction fitness for seed thickness.

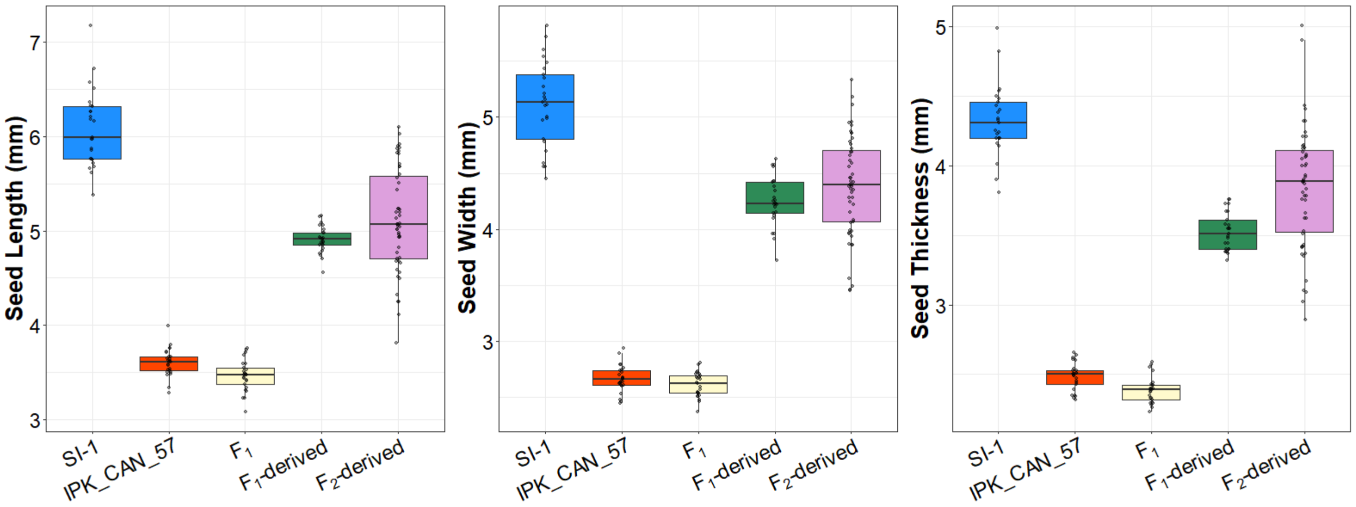

(a)

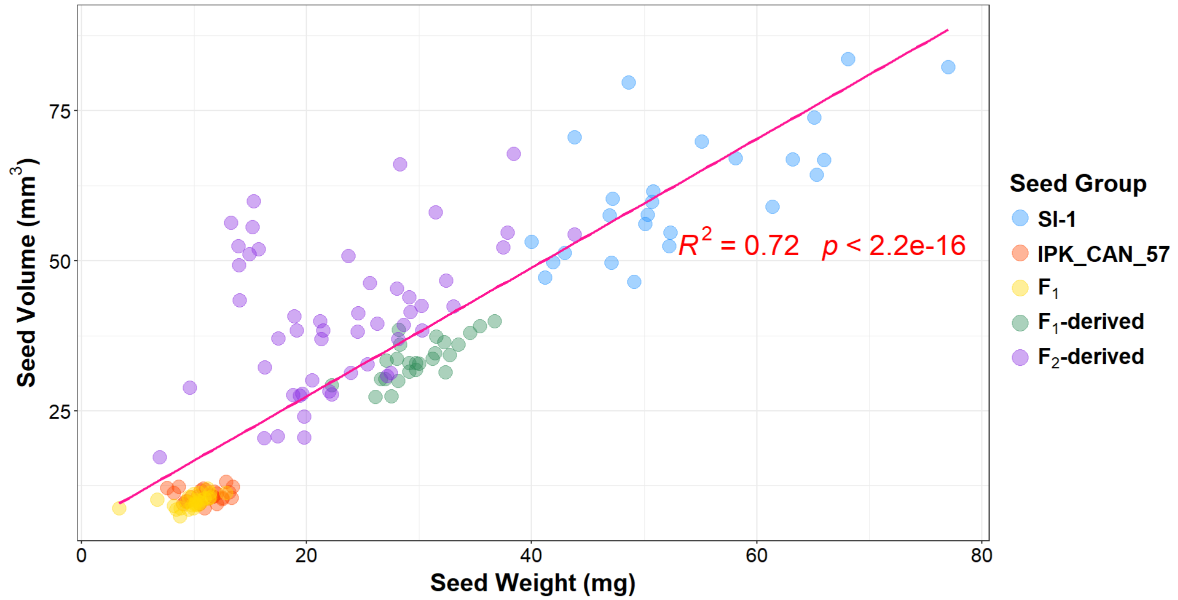

(b)

Figure S5. (a) Seed traits measured for SI-1 (n=25), IPK_CAN_57 (n=25), F_1 (n=25), F_1-derived (n=25), and F_2-derived seeds (n=51); (b) Correlation between seed weight and seed volume based on SI-1 (n=25), IPK_CAN_57 (n=25), F_1 (n=25), F_1-derived (n=25), and F_2-derived seeds (n=51).

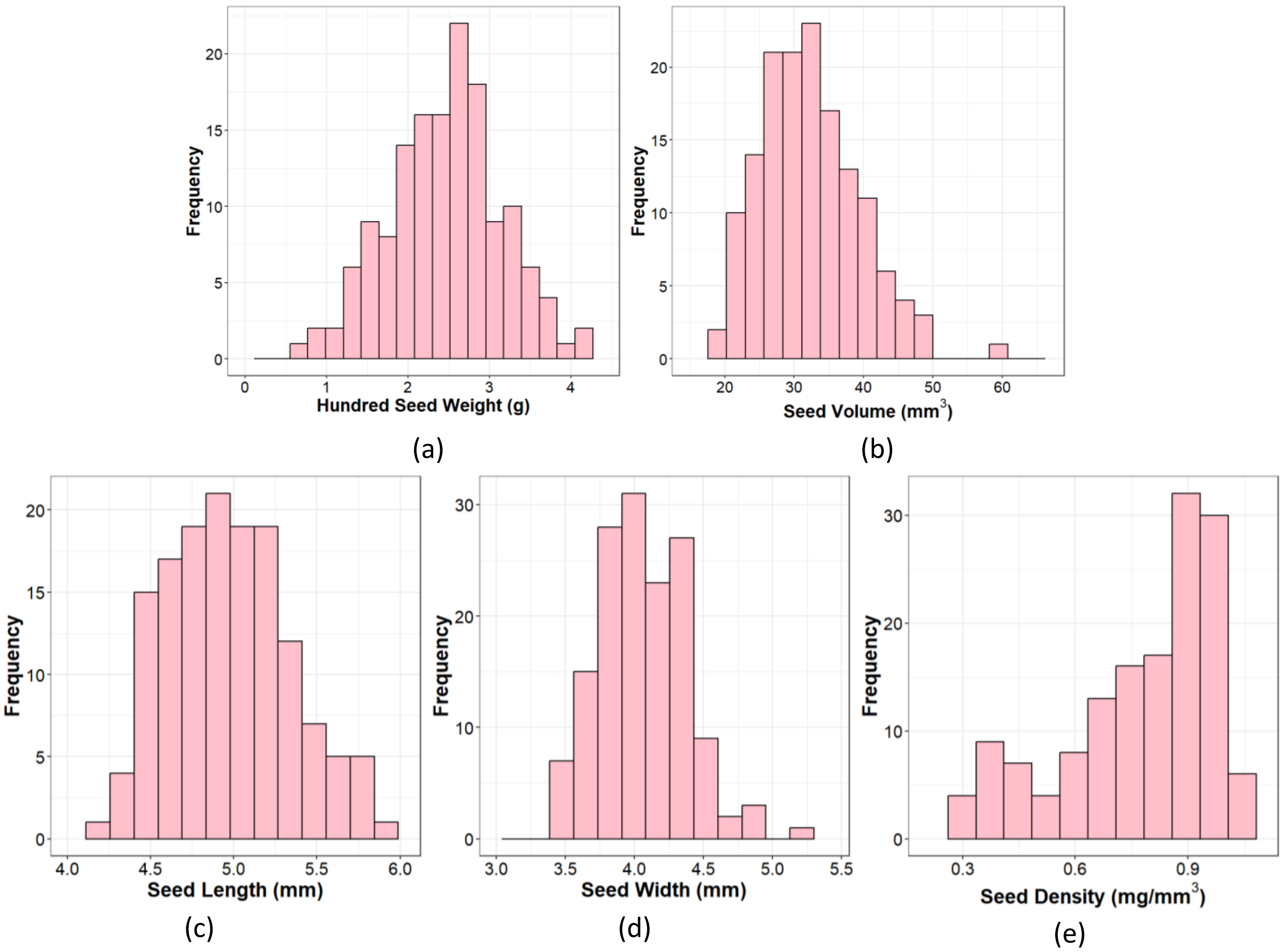

Figure S6. Distribution of seed traits – (a) hundred seed weight, (b) seed volume, (c) seed length, (d) seed width, and (e) seed density – of the F_2-derived seeds generated from the cross between SI-1 and IPK_CAN_57 (n=147).

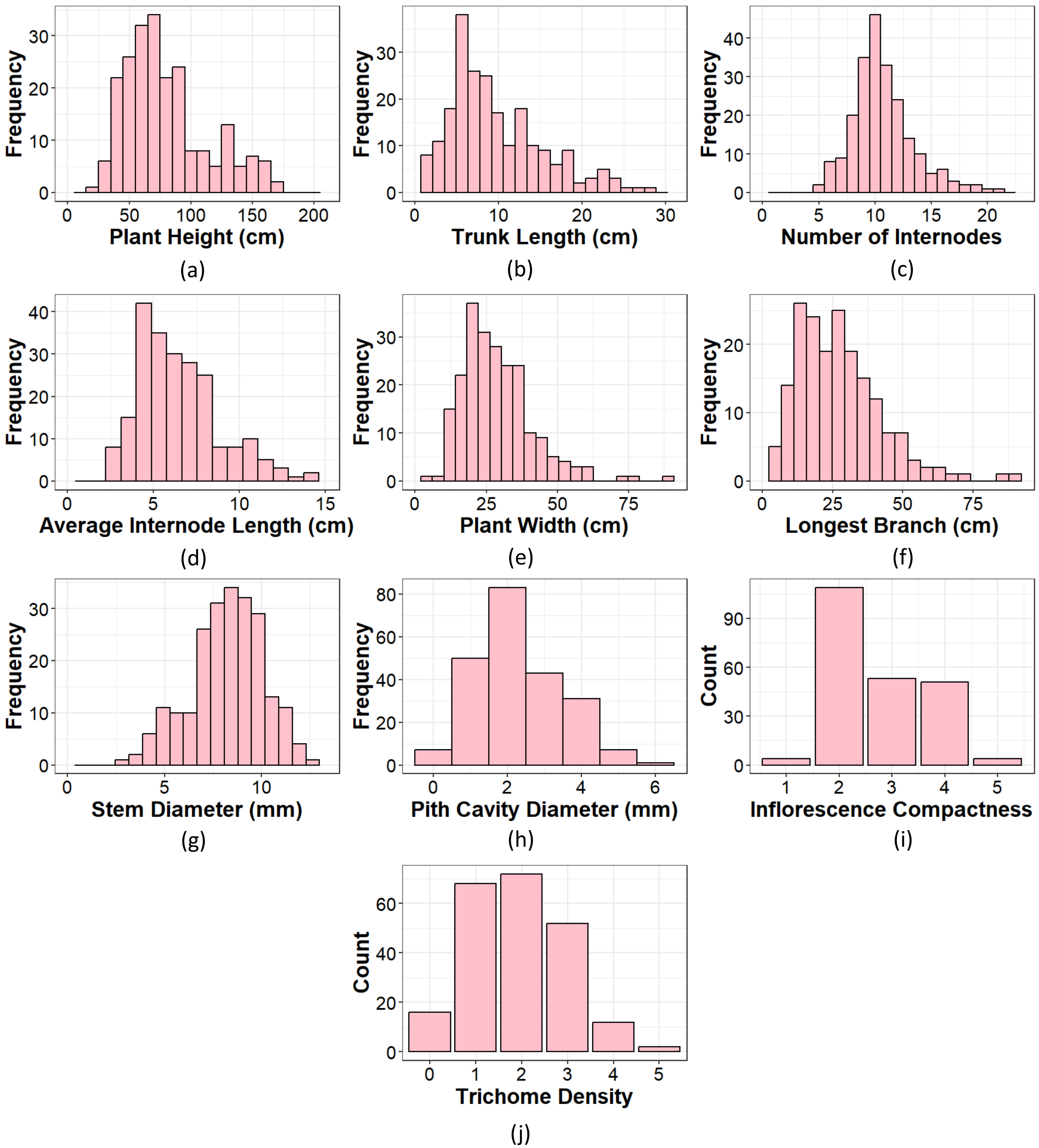

Figure S7. Distribution of agronomic traits – (a) plant height, (b) trunk length, (c) number of internodes, (d) average internode length, (e) plant width, (f) longest branch, (g) stem diameter, (h) pith cavity diameter, (i) inflorescence compactness, and (j) trichome density – of the F_2 population generated from the cross between SI-1 and IPK_CAN_57 (n=222).

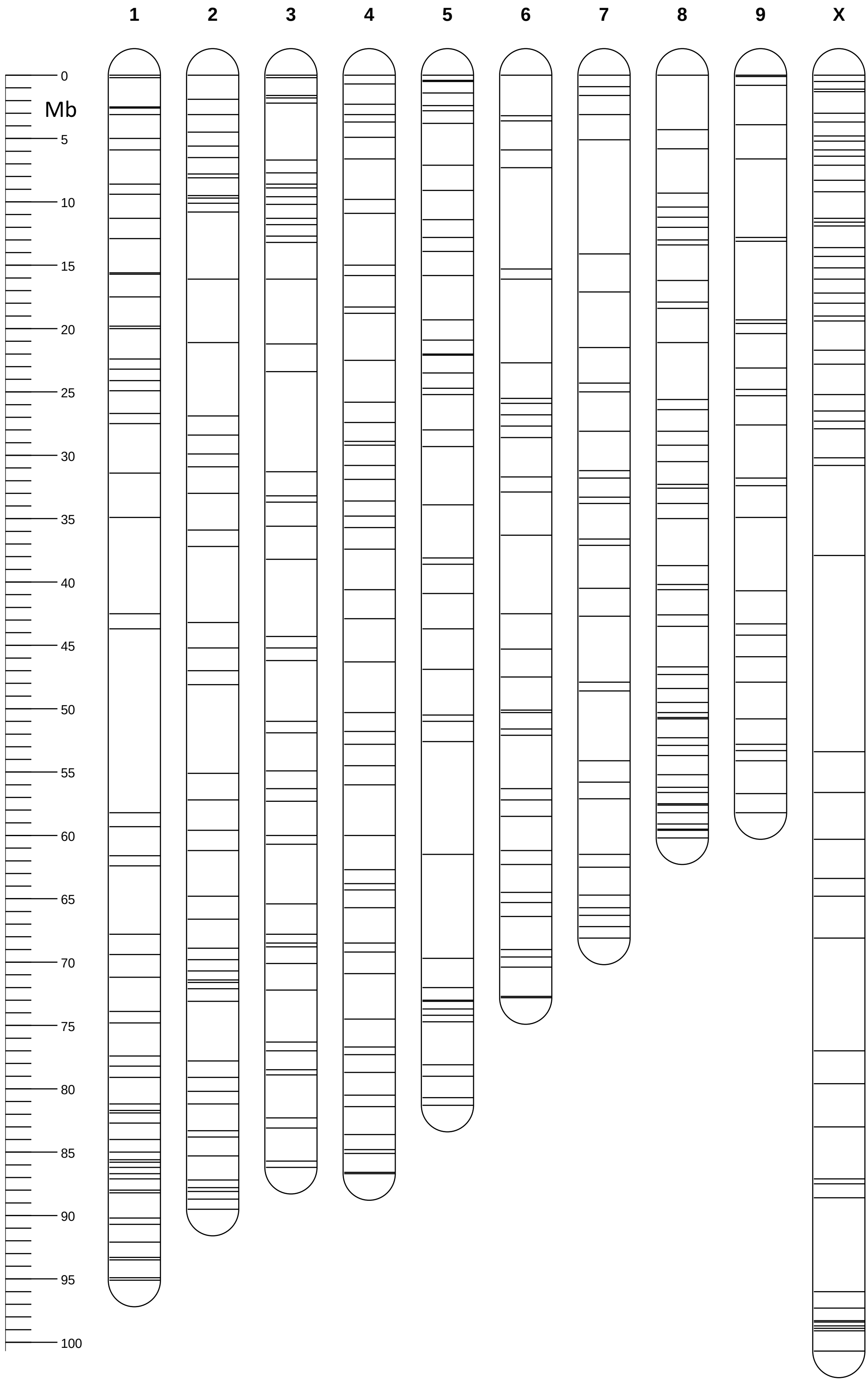

Figure S8. The physical positions of the 455 genetic markers used in this study.

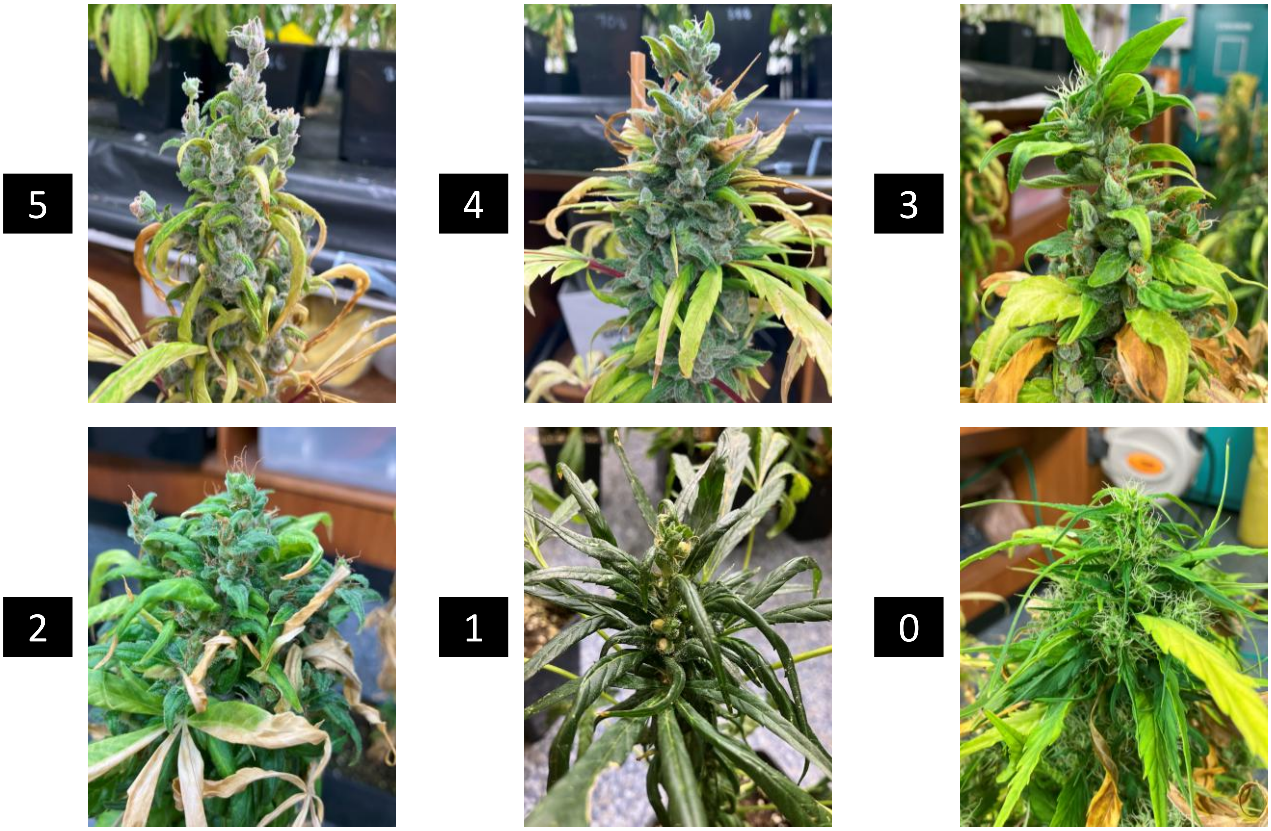

Figure S9. Trichome density scale followed in this study based on varying degrees of trichome density of six plants selected from the F_2 population.

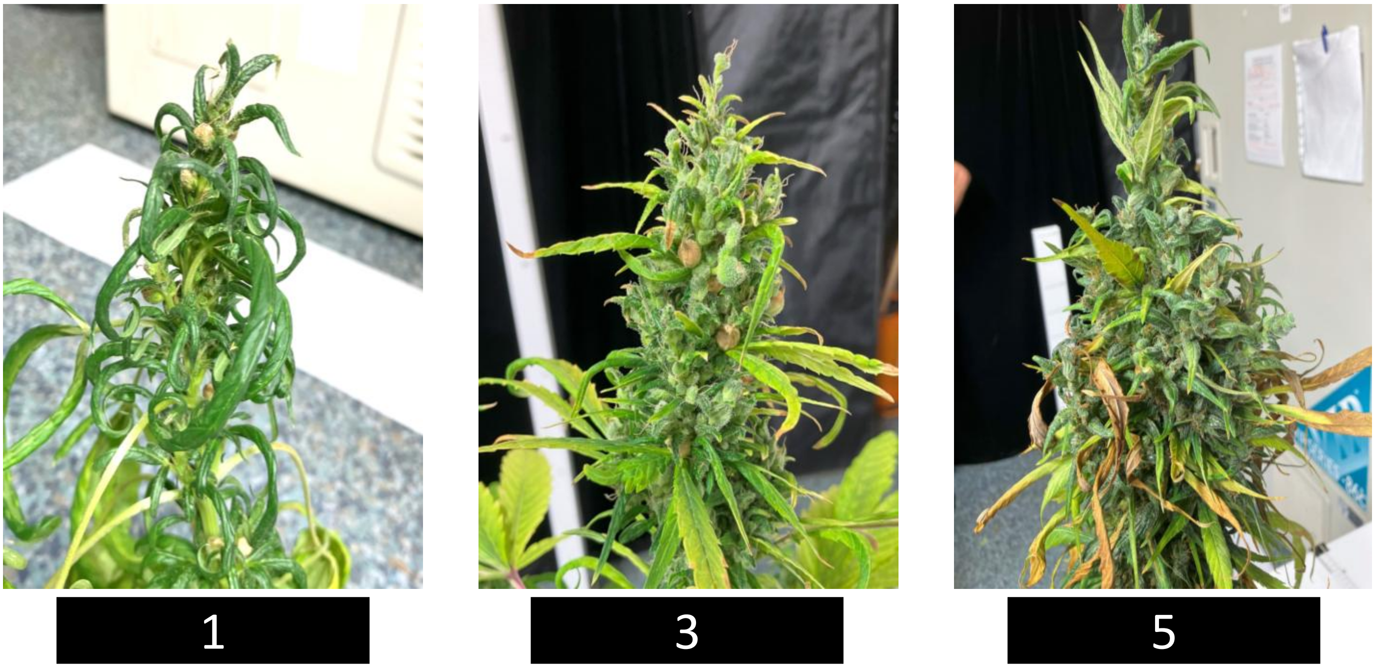

Figure S10. Inflorescence compactness scale followed in this study based on varying degrees of inflorescence compactness of three plants selected from the F_2 population.
